# SIRT1-mediated deacetylation of FOXO3 enhances mitophagy and drives hormone resistance in endometrial cancer

**DOI:** 10.1186/s10020-024-00915-7

**Published:** 2024-09-12

**Authors:** Xuehua Wei, Xiangpeng Xiong, Pingping Wang, Shufang Zhang, Dongxian Peng

**Affiliations:** 1 Obstetrics and Gynecology Center, Department of Gynecology, Zhujiang Hospital, Southern Medical University, No. 253, Industry Avenue, Haizhu District, Guangzhou, 510280 Guangdong China; 2 Department of Gynecology, Jiangxi Maternal and Child Health Hospital, Nanchang, 336000 China; 3 Department of Gynecology, Southern University of Science and Technology Hospital, Shenzhen, 518000 China

**Keywords:** Endometrial cancer, SIRT1, FOXO3, Mitophagy, Hormone resistance, Deacetylation modification, BNIP3/PINK1/Parkin signaling pathway

## Abstract

**Background:**

The complex interplay between Sirtuin 1 (SIRT1) and FOXO3 in endometrial cancer (EC) remains understudied. This research aims to unravel the interactions of deacetylase SIRT1 and transcription factor FOXO3 in EC, focusing on their impact on mitophagy and hormone resistance.

**Methods:**

High-throughput sequencing, cell experiments, and bioinformatics tools were employed to investigate the roles and interactions of SIRT1 and FOXO3 in EC. Co-immunoprecipitation (Co-IP) assay was used to assess the interaction between SIRT1 and FOXO3 in RL95-2 cells. Functional assays were used to assess cell viability, proliferation, migration, invasion, apoptosis, and the expression of related genes and proteins. A mouse model of EC was established to evaluate tumor growth and hormone resistance under different interventions. Immunohistochemistry and TUNEL assays were used to assess protein expression and apoptosis in tumor tissues.

**Results:**

High-throughput transcriptome sequencing revealed a close association between SIRT1, FOXO3, and EC development. Co-IP showed a protein–protein interaction between SIRT1 and FOXO3. Overexpression of SIRT1 enhanced FOXO3 deacetylation and activity, promoting BNIP3 transcription and PINK1/Parkin-mediated mitophagy, which in turn promoted cell proliferation, migration, invasion, and inhibited apoptosis in vitro, as well as increased tumor growth and hormone resistance in vivo. These findings highlighted SIRT1 as an upstream regulator and potential therapeutic target in EC.

**Conclusion:**

This study reveals a novel molecular mechanism underlying the functional relevance of SIRT1 in regulating mitophagy and hormone resistance through the deacetylation of FOXO3 in EC, thereby providing valuable insights for new therapeutic strategies.

**Supplementary Information:**

The online version contains supplementary material available at 10.1186/s10020-024-00915-7.

## Introduction

Endometrial cancer (EC) is a highly prevalent gynecological malignancy, especially in developed countries (Kailasam and Langstraat 2022; Kalampokas et al. 2022; Contreras et al. 2022). In recent years, there has been an increasing incidence of EC due to changes in lifestyle and an aging population (Crosbie et al. 2022). Treatment options for EC are diverse, including surgery, radiation therapy, chemotherapy, and hormone therapy (Rütten et al. 2021). However, treatment efficacy is often limited by the problem of hormone resistance, which significantly hampers the effectiveness of hormone therapy (Gordhandas et al. 2023). Hormone resistance primarily affects the response to hormone therapy and involves various cellular and molecular mechanisms (Beaupere et al. 2021). While it does not directly impact the mechanisms of radiation therapy or chemotherapy, multidrug resistance (MDR) phenomena may influence overall treatment outcomes (Duan et al. 2023). Therefore, it is of great significance to study the pathogenesis of EC, particularly the molecular mechanisms associated with hormone resistance, to improve treatment strategies for EC (Knez et al. 2021).

Autophagy is a self-degradation process crucial for balancing energy sources and managing nutritional stress during critical developmental periods (Yang et al. 2024; Ashrafizadeh et al. 2023). Autophagy also plays a housekeeping role by removing misfolded or aggregated proteins, damaged organelles (e.g., mitochondria, endoplasmic reticulum, and peroxisomes), and intracellular pathogens (Qin et al. 2023; Glick et al. 2010). Mitophagy, a specialized form of autophagy, is essential for maintaining cellular homeostasis and adapting to stress responses (Onishi et al. 2021). Recent studies have shown that mitophagy plays a significant role in the occurrence and development of cancers (Xu and Hu 2022; Panigrahi et al. 2020). Aberrant mitophagy activity in EC is closely associated with tumor cell metabolism, proliferation, survival, and resistance (Song et al. 2022). For instance, dysregulation of mitophagy can lead to abnormal cell metabolism, thereby promoting tumor cell proliferation and survival (Praharaj et al. 2022; Panigrahi et al. 2020). Additionally, changes in mitophagy are related to the sensitivity of EC cells to chemotherapy drugs and hormone therapy (Fukuda and Wada-Hiraike 2022). Therefore, a comprehensive understanding of the role of mitophagy in EC may provide important insights for developing new treatment strategies.

Sirtuin 1 (SIRT1), a deacetylase, is a key regulatory factor in cellular energy sensing and stress responses (Yang et al. 2022; Singh and Ubaid 2020). It participates in the regulation of cellular metabolism, DNA repair, cell cycle control, apoptosis, and other processes by deacetylating a variety of substrate proteins (Garcia-Peterson and Li 2021; Sinha et al. 2020). FOXO3, as a member of the forkhead box transcription factor family, plays a vital role in regulating cell apoptosis, antioxidant stress, and the cell cycle (Jerome et al. 2022; Jiramongkol and Lam 2020). The altered expression and activity of SIRT1 and FOXO3 are closely associated with tumorigenesis and tumor progression in various cancers (Dilmac et al. 2022; Farhan et al. 2020). However, their functions and regulatory mechanisms in EC are not fully understood. Therefore, exploring the roles of SIRT1 and FOXO3 in EC is of great significance for understanding the pathogenesis of EC and developing new therapeutic approaches.

The interaction between SIRT1 and FOXO3 is crucial for maintaining cellular homeostasis (Cheng 2022; Chen et al. 2020). In EC, SIRT1 can deacetylate FOXO3, affecting its transcriptional activity and subsequently regulating various downstream biological processes (Rezk et al. 2021; Jiramongkol and Lam 2020; Halasa et al. 2021). For example, by activating FOXO3, SIRT1 may influence the autophagy process, proliferation, migration, and invasion capacity of EC cells, as well as their response to hormone therapy (Kratz et al. 2021; Wang et al. 2022). Moreover, the interaction between SIRT1 and FOXO3 may also play a role in regulating the sensitivity of EC cells to chemotherapy drugs (Yousafzai et al. 2021; Garcia-Peterson and Li 2021). Therefore, a deep investigation into the interaction between SIRT1 and FOXO3 in EC is significant for understanding the molecular mechanisms of EC and developing targeted therapeutic strategies.

This study aims to explore the roles, interactions, and effects of SIRT1 and FOXO3 in EC, specifically on mitophagy and hormone resistance (Figure S1). The molecular mechanisms of SIRT1 and FOXO3 in the development of EC were revealed through cellular experiments, animal models, and bioinformatics analysis. SIRT1 and FOXO3 regulated the growth, migration, and drug resistance of EC cells via mitophagy. Additionally, the study evaluated the potential of SIRT1 and FOXO3 as therapeutic targets, providing a theoretical basis for developing novel treatments for EC. The findings may enhance the prevention, diagnosis, and treatment of EC, addressing hormone resistance and offering more effective therapeutic options for patients.

## Materials and methods

### RNA extraction

EC cells were cultured to 80–90% confluency and then digested with 0.25% trypsin–EDTA (25,200,056, Thermo Fisher, USA) for 3–5 min. Digestion was halted with a culture medium containing 10% FBS (10100147C, Thermo Fisher, USA). Cells were collected by centrifugation at 1000 rpm for 5 min, and the supernatant was discarded. Total RNA was extracted using the Trizol reagent kit (A33254, Thermo Fisher, USA) according to the manufacturer's instructions. The RNA precipitate was washed with 75% ethanol (E299585, Aladdin, Shanghai, China), air-dried for 5–10 min, and dissolved in DEPC-treated water (Akbar et al. 2019). The purpose of RNA extraction was to obtain high-quality RNA for subsequent high-throughput transcriptome sequencing.

The purity and integrity of RNA were assessed using a Nanodrop ND-1000 spectrophotometer (Thermo Fisher) by measuring the OD260/280 ratio, ensuring no protein or organic contamination. RNA concentration was measured with the Qubit RNA assay kit (Q33221, Thermo Fisher, USA). RNA samples meeting the criteria of RNA integrity number (RIN) ≥ 7.0, 28S:18S ratio ≥ 1.5 were used for further experiments (Gonye et al. 2023).

### High-throughput transcriptome sequencing

Sequencing libraries were prepared and sequenced by CapitalBio Technology (Beijing, China) using 5 μg of RNA per sample. Ribosomal RNA (rRNA) was removed from total RNA using the Ribo-Zero Magnetic Kit (MRZG12324, Epicentre, USA). The Illumina NEB Next Ultra RNA Library Prep Kit (E7760S, NEB, USA) was used for library construction. RNA fragments were then fragmented into approximately 300 base pair (bp) segments in NEB Next First Strand Synthesis Reaction Buffer (5×). First-strand cDNA was synthesized using reverse transcriptase and random primers, followed by second-strand cDNA synthesis in dUTP Mix (10×) Second Strand Synthesis Reaction Buffer. The ends of the cDNA fragments were repaired, including the addition of A-tails and adapter ligation. After ligating the Illumina sequencing adapters, the second strand cDNA was digested using the USER enzyme (M5508, NEB, USA) to construct a strand-specific library. The library DNA was amplified, purified, and enriched by PCR. The library was validated using an Agilent 2100 instrument and quantified with the KAPA Library Quantification Kit (kk3605, Merck, USA). Finally, paired-end sequencing was performed on the Illumina NextSeqCN500 platform (Ayturk 2019; Simoneau et al. 2021). This experiment was undertaken to identify differentially expressed genes (DEGs), and the results guided subsequent functional analyses.

### Transcriptome sequencing data analysis

The quality of the raw sequencing paired-end reads was assessed using FastQC software v0.11.8. The initial data were preprocessed with Cutadapt software v1.18 to remove Illumina sequencing adapters and poly(A) tails. Reads containing more than 5% ambiguous bases (N) were discarded using Perl scripts. The FASTX Toolkit v0.0.13 was employed to retain reads, with at least 70% of bases having a quality score above 20. BBMap software was used to repair paired-end sequences. Finally, high-quality filtered reads were aligned to the human reference genome using hisat2 software v0.7.12. This preprocessing step ensured the data quality for subsequent differential expression analysis.

Differential expression analysis of mRNA read counts was conducted using the "edgeR" package in R, with criteria set at |log2FC|> 1 and *p* < 0.05. This step aimed to identify genes significantly associated with EC progression and provided a basis for further functional analyses.

Autophagy-related genes were identified from the GeneCards database (https://www.genecards.org/) using the search term "Autophagy," resulting in 327 relevant genes. A Venn analysis was performed using the "VennDiagram" package in R to intersect these genes with the DEGs identified earlier. This intersection yielded significant autophagy-related DEGs (Stelzer et al. 2016).

A heatmap of the intersecting genes was generated using the "heatmap" package in R. Protein–protein interaction (PPI) analysis of key factors was performed using the STRING database (https://string-db.org/) with a minimum interaction score of 0.700. The interaction networks were visualized with Cytoscape v3.5.1, and core genes were identified using the CytoHubba tool.

The "ClusterProfiler" package in R was used for Gene Ontology (GO) functional enrichment analysis of the intersecting genes, covering biological processes (BP), molecular functions (MF), and cellular components (CC). Results were visualized with bubble and circle plots, using *P* < 0.05 as the threshold. Additionally, the Kyoto Encyclopedia of Genes and Genomes (KEGG) pathway enrichment analysis was performed on candidate targets using the "ClusterProfiler" package, with visualizations in bubble plots and circle plots (Shi et al. 2020).

### TCGA data analysis for survival analysis

RNA-seq data (FPKM format) and clinical data for the TCGA-UCEC (Uterine Corpus EC) project (n = 553) were downloaded from the TCGA database (https://portal.gdc.cancer.gov). Samples lacking clinical information or normal samples were excluded. Proportional hazard assumptions were tested, and survival regressions were fitted using the "survival" package in R. The results were visualized with the "survminer" and "ggplot2" packages in R (Liu et al. 2018). This survival analysis aimed to determine the prognostic significance of SIRT1 and FOXO3 in EC, further supporting their potential as therapeutic targets.

### Cell culture and in vitro experimental protocols

The human embryonic kidney cells (HEK-293T) and EC cells (Ishikawa, RL95-2, KLE, AN3CA, and HHUA) were all purchased from Biobw Biotechnology Co., Ltd. (Bio-72947, Bio-73224, Bio-73138, Bio-73074, Bio-53662, and Bio-133241; Beijing, China). Human endometrial cells (EEC) were obtained from KeyCell Biotechnology Co., Ltd. (QS-H011, Wuhan, China) and cultured in MEM medium supplemented with non-essential amino acids (NEAA) (PM150410, Procell Co., Ltd.; Wuhan, China) containing 10% FBS and 1% antibiotics (100 U/mL penicillin and 100 μg/mL streptomycin, 15,140,163, Thermo Fisher, USA). The remaining cell lines were cultured in high-glucose DMEM medium (11,965,084, Thermo Fisher, USA) supplemented with 10% FBS and 1% antibiotics. All cells were maintained in a humidified CO_2_ incubator (Heracell™ Vios 160i CR CO_2_ incubator, 51,033,770, Thermo Fisher) at 37 °C with 5% CO_2_. Cells were passaged when they reached 80–90% confluency (Asaka et al. 2015).

For cell treatments, cells were seeded in 6-well plates and allowed to adhere overnight. Then, a deacetylase inhibitor cocktail (DIC) (P1112, Beyotime, Shanghai, China) was added to the cell culture medium at a 1:100 ratio following the manufacturer's instructions for 24 h of treatment (Zheng et al. 2022). Alternatively, cells were treated with 10 μM of the autophagy inhibitor chloroquine (CQ, HY-17589A, MedChemExpress, USA) for 24 h before subsequent experiments (Xu et al. 2024). These treatments aimed to investigate the effects of deacetylase inhibition and autophagy inhibition on EC cells.

For cell grouping, cells were divided into the following groups to explore the interactions between SIRT1 and FOXO3 and their effects on EC cell behavior: negative control (NC) of short hairpin RNA (shRNA, sh-NC), shRNA targeting SIRT (sh-SIRT1), overexpression (oe)-NC, oe-SIRT1, sh-NC, sh-FOXO3, oe-NC + dimethyl sulfoxide (DMSO), oe-NC + DIC, oe-SIRT1 + DMSO, oe-SIRT1 + DIC, oe-SIRT1 + DMSO, oe-SIRT1 + CQ, oe-NC + sh-NC, oe-SIRT1 + sh-NC, oe-SIRT1 + sh-FOXO3.

### Lentivirus and plasmid transduction

Lentiviral infection was employed to overexpress or silence genes in EC cells, with lentiviral packaging services provided by Sangon Biotech, Shanghai, China. Plasmids from the pHAGE-puro series and auxiliary plasmids pSPAX2 and pMD2.G (Addgene, USA, catalog numbers #118,692, #12,260, and #12,259) were co-transfected into HEK293T cells along with pSuper-retro-puro series plasmids and auxiliary plasmids gag/pol and VSVG (catalog numbers #113,535, #14,887, and #8454). After 48 h of cell culture, the supernatant containing lentiviral particles was collected and filtered through a 0.45 μm filter. A second collection was performed at 72 h, and the viral particles were concentrated by centrifugation. The two viral harvests were combined, and viral titers were determined.

The logarithmic-phase cells were digested with trypsin and seeded at a density of 1 × 10^5^ cells per well in a 6-well plate. After 24 h of routine culture, when the cell confluence reached approximately 75%, cells were infected with lentiviral particles (MOI = 10, working titer approximately 5 × 10^6^ TU/mL) and 5 μg/mL polybrene (TR-1003, Merck, USA). After 4 h, the medium was diluted with an equal volume of culture medium to reduce polybrene concentration. The medium was replaced with fresh culture medium 24 h post-infection.

Cells were selected with puromycin (E607054, Sangon Biotech) to construct stably transfected cell lines at an appropriate concentration. Lentiviral silencing sequences are shown in Table S1, and the sequences with the best silencing efficiency were used for subsequent experiments.

### RT-qPCR

Total RNA from cells was extracted using the Trizol reagent kit, and cDNA was synthesized using the PrimeScript RT Reagent Kit (RR047A, Takara, Japan). RT-qPCR was performed using the SYBR^®^ Premix Ex TaqTM II kit (DRR081, Takara, Japan) on an ABI7500 real-time PCR system (Thermo Fisher, USA). GAPDH was used as the internal control, and each RT-qPCR experiment included three technical replicates. The relative expression of target genes was calculated using the 2^−ΔΔCt^ formula (Ayuk et al. 2016). Primer sequences are detailed in Table S2.

### Western blot

Cells were lysed using RIPA lysis buffer containing 1% PMSF (P0013B, Beyotime) to extract total protein, following the manufacturer's instructions. The total protein concentration of each sample was determined using a BCA Protein Assay Kit (P0011, Beyotime) and adjusted to 1 μg/μL. Based on the target protein size, sodium dodecyl sulfate–polyacrylamide gel electrophoresis (SDS-PAGE; 8–12%) was prepared. Next, 50 μg of protein samples were loaded into each lane. The proteins on the gel were then transferred onto a PVDF membrane (1,620,177, Bio-Rad, USA). The membrane was blocked with 5% non-fat milk in 1 × TBST at room temperature for 1 h. The membrane was incubated overnight at 4 °C with the primary antibodies (antibody information provided in Table S3). Subsequently, the membrane was then incubated at room temperature for 1 h with HRP-conjugated goat anti-rabbit IgG (ab6721, Abcam, Cambridge, UK, 1:5000) or goat anti-mouse IgG (ab205719, Abcam, 1:5000) secondary antibody. The blots were visualized in ECL reaction solution (1,705,062, Bio-Rad, USA), and the bands were imaged using an Image Quant LAS 4000C gel imaging system (GE, USA). The grayscale values of the target bands were normalized to the internal reference band GAPDH (Walentowicz-Sadlecka et al. 2019).

### Co-immunoprecipitation (Co-IP)

The Co-IP experiment was performed to detect PPIs in RL95-2 cells, particularly between SIRT1 and FOXO3. RL95-2 cells were lysed on ice for 10 min using an IP lysis buffer containing protease and phosphatase inhibitors (P0013, Beyotime). Lysates were centrifuged at 12,000*g* for 20 min at 4 °C, and the supernatant was collected. Twenty microliters of the lysate were set aside as input, and the remainder was incubated with 10 µL of Protein G magnetic beads (10004D, Thermo Fisher, USA) and 1 µL of anti-SIRT1 (2493S, Cell Signal Technology, USA) or anti-FOXO3 (12829S, Cell Signal Technology, USA) antibodies. The mixture was incubated on a shaker overnight at 4 ℃. The immunocomplexes were then washed four times with NETN buffer NETN buffer (20 mM Tris, pH 8.0, 100 mM NaCl, 1 mM EDTA, and 0.5% NP-40), separated by SDS-PAGE, and analyzed by Western blot using appropriate antibodies (Che et al. 2020).

### SIRT1 activity assay

The SIRT1 enzyme activity was measured using the SIRT1 enzyme activity assay kit (ab156065, Abcam) according to the manufacturer's instructions. In each well of a microplate, 25 µL of HPLC-grade H_2_O, 5 µL of SIRT1 detection buffer, 5 µL of fluorescent substrate peptide, and 5 µL of NAD were added. Then, 5 µL of the sample was added to each well. The reaction was initiated by adding 5 µL of developing reagent to each well at 24.0 ± 2.0 ℃, followed by thorough mixing. The fluorescence intensity was measured using a microplate fluorometer (SpectraMax M3, USA) with an excitation wavelength of 360 nm and an emission wavelength of 485 nm. The measurements were taken every 2 min for a total duration of 60 min. The SIRT1 enzyme activity of the extract was calculated using the following formula (Salee et al. 2022):

$$SIRT1 activity=(\frac{{FI}_{sample}}{{FI}_{control}})\times 100\%$$

FI_sample_ and FI_control_ represented the fluorescence intensities of the sample and the control solution, respectively. The enzymatic activity of SIRT1 was expressed as the ratio of fluorescence intensity (Salee et al. 2022). This assay was used to assess the functional activity of SIRT1 in EC cells and delineate its role in mitophagy and hormone resistance.

### CCK-8 cell viability assay

Cell concentration was adjusted to 1 × 10^3^ cells/mL and then seeded into a 96-well plate with a volume of 100 μL per well. Cell viability was assessed at 12, 24, 36, and 48 h using the CCK-8 kit (C0041, Beyotime), following the manufacturer’s instructions. Subsequently, 10 μL of CCK-8 solution was added to each well and incubated at 37 ℃ and 5% CO_2_ for 2 h. The absorbance at 450 nm was measured using an ELISA reader to calculate cell viability (Liu et al. 2021).

### EdU staining assay

EC cells were seeded in 24-well plates at a density of 1 × 10^5^ cells per well, with three replicates per group. EdU solution (ST067, Beyotime) was added to the medium at a final concentration of 10 µmol/L and incubated for 2 h. Cells were then fixed with 4% paraformaldehyde in PBS for 15 min at room temperature, washed twice with PBS containing 3% BSA, and permeabilized with 0.5% Triton-100 in PBS for 20 min. Next, 100 µL of staining solution was added to each well, and the cells were incubated at room temperature in the dark for 30 min. Then, DAPI (C1002, Beyotime) was added to stain the cell nuclei for 5 min. The percentage of EdU-positive cells was calculated under a fluorescence microscope (FM-600, Shanghai Putian Optical Instrument Co., Ltd.) by observing 6–10 random fields per well (Yu et al. 2020). This assay was used to asses cell proliferation and determine the effects of gene modulation on cell cycle progression.

### Colony formation assay

EC cells were seeded into each well of a six-well plate and cultured for 2 weeks, with the medium changed every 3 days. Colonies were fixed with methanol for 20 min and stained with 0.1% crystal violet (C0121, Beyotime) for 15 min. After rinsing, colonies were photographed and analyzed using Image Pro Plus 6.0 software (Liu et al. 2021). This assay provided insights into the clonogenic potential of treated cells and informed further investigations.

### Transwell assay

Transwell invasion assays were performed after 24 h of different treatments. Transwell chambers were coated with 50 µL of Matrigel (354,234, BD Biosciences, USA) and incubated at 37 °C for 30 min to solidify. Cells were diluted to 2.5 × 10^4^ cells/mL, and 100 µL of cell suspension was added to the upper chamber, while 500 µL of medium containing 10% FBS was added to the lower chamber. After 24 h, the cells on the upper surface of the membrane were removed with a cotton swab, and the cells that had invaded through the membrane were fixed with 4% paraformaldehyde for 30 min, stained with 0.1% crystal violet for 30 min, and photographed under an inverted microscope (IXplore Pro, Olympus, Japan). Five random fields were counted for each well (Fan et al. 2020). The migration assay followed the same steps but without Matrigel coating. These assays were performed to evaluate the invasive and migratory abilities of treated cells.

### Wound healing assay

Lines were drawn at the bottom of a six-well plate using a ruler and marker at intervals of 0.5–1 cm, with at least five lines per well. EC cells were seeded at a density of 5 × 10^5^ cells per well. When the cells reached 100% confluency, scratches were made perpendicular to the drawn lines using a 200 µL pipette tip. The medium was replaced with serum-free medium, and images of the scratches were captured at 0 and 24 h using an optical microscope (DM500, Leica). The distance between the wound edges was measured using ImageJ software, and the wound healing rate was calculated using the following formula (Liu et al. 2021):

$$Wound healing rate=\frac{{{distance}_{0 h}-distance}_{24 h}}{{distance}_{0 h}}$$

"distance_0 h_" and "distance_24 h_" represent the distances between the cell scratches at 0 h and 24 h after scratching, respectively. This assay was conducted to evaluate cell migration and inform further studies on cell motility.

### Flow cytometry

Apoptosis in EC cells was detected using the Annexin V-FITC/PI apoptosis detection kit (C1062L, Beyotime). Cells were seeded at 1 × 10^6^ cells per well in six-well plates. After treatment, cells were collected, resuspended in 195 µL Annexin V-FITC binding buffer, and incubated with 5 µL Annexin V-FITC and 10 µL PI solution for 15 min in the dark. Flow cytometry analysis was performed within 20 min using a BD FACS Calibur flow cytometer to determine apoptosis rates (Liu et al. 2021).

Mitochondrial membrane potential (MMP) was assessed using the JC-1 kit (KGA604, Jiangsu KeyGen Biotech, China) according to the manufacturer's instructions. Briefly, 1 × 10^6^ EC cells were resuspended in 500 μL of JC-1 staining working solution and incubated at 37 °C for 20 min. Cells were centrifuged at 2000 rpm for 5 min and resuspended in 500 μL of 1 × Incubation buffer, and analyzed by flow cytometry (excitation: 488 nm, emission: 530 nm) (Yao et al. 2022). These assays were adopted to elucidate the impact of treatments on cell apoptosis and mitochondrial function.

### Transmission electron microscopy (TEM)

To observe EC cells under TEM, cells were first fixed in 3% glutaraldehyde (49,629, Sigma, USA) in 0.1 M phosphate buffer (pH 7.4, 17,202, Sigma, USA), followed by post-fixation with 1% osmium tetroxide (OsO_4_, O5500, Sigma, USA). After dehydration, 10 nm thick sections were prepared and stained with uranyl acetate (SPI-02624, Beijing Haide Chuangye Biotechnology, China) and lead nitrate (NIST928, Sigma, USA). Images were captured at 80 kV using a Hitachi H7650 TEM (Hitachi, Japan). Five random fields were selected for quantitative analysis of autophagic vacuoles (Lin et al. 2020). This step was carried out to visually confirm the presence of autophagic structures.

### GFP-LC3B plasmid transfection

RL95-2 cells (1 × 10^5^ cells per well) were seeded in a 24-well plate, with three replicates per group. After overnight incubation for cell adhesion, the GFP-LC3B plasmid (D2815, Beyotime) was transfected into RL95-2 cells using lentivirus. Following a 12-h transfection period, cells were fixed with 4% paraformaldehyde (P885233, Macklin, Shanghai, China) for 10 min. Cells were then stained with DAPI (#4083, Cell Signaling Technology, USA) for 10 min and mounted with 20 μL of mounting medium. Green fluorescent puncta were observed under a fluorescence microscope (Zeiss Observer Z1, Germany) (Chen et al. 2022). This experiment was conducted to monitor autophagosome formation.

### Mitochondrial reactive oxygen species (ROS) detection

EC cells were digested, resuspended, and adjusted to a concentration of 1 × 10^6^ cells/mL. Then, cells were plated (2 mL/well) into six-well plates and cultured overnight. Cells or tissues were washed with PBS and incubated with 5 μL of MitoSOX mitochondrial superoxide indicator (M36008, Thermo Fisher, USA) in PBS at 37 ℃ in the dark for 10 min. After washing with PBS to remove excess MitoSOX reagent, cells or tissues were fixed with 4% paraformaldehyde and stained with DAPI for 10 min. After three washes with PBS, cells were mounted with 20 μL of mounting medium and examined using a fluorescence microscope (Robinson et al. 2008). This assay was employed to assess mitochondrial ROS levels, informing further studies on oxidative stress in EC cells.

### Immunofluorescence co-localization for Mitophagy

EC cells were digested, resuspended, and adjusted to a concentration of 1 × 10^6^ cells/mL. Then, cells were plated (2 mL/well) into six-well plates and cultured overnight. After removing the culture medium, cells were washed twice with PBS. Mitochondrial selective probe Mitotracker Green FM (M7514, Thermo Fisher, USA) and lysosomal selective probe Lysotracker^®^ Red DND-99 (L7528, Thermo Fisher, USA) were added to the cells at final concentrations of 0.1 µM and 0.025 µM, respectively, and incubated in the dark for 30 min. Cells were fixed with 4% paraformaldehyde and stained with DAPI for 10 min. Then, cells were mounted with 20 µL of mounting medium and examined using a fluorescence microscope (Shida et al. 2016; Li et al. 2017). This experiment was designed to visualize and quantify mitophagy by co-localizing mitochondria and lysosomes.

### Cell immunofluorescence

Cells were fixed with 4% paraformaldehyde for 15–30 min and permeabilized with 0.1% Triton (L885651, Macklin) for 15 min. Next, cells were blocked with PBS containing 15% FBS at 5 °C overnight.

Cells were incubated with rabbit anti-FOXO3 antibody (MA5-14,932, 1:200, Thermo Fisher, USA) at 37 °C for 60 min. Subsequently, cells were incubated with FITC-conjugated goat anti-rabbit secondary antibody at 37 °C in the dark for 60 min. Finally, cells were stained with Alexa-488 conjugated goat anti-rabbit antibody (ab150129, Abcam) and DAPI for 1 h at room temperature. Cells were mounted with 20 μL of mounting medium for observation under a fluorescence microscope (Wagle et al. 2016). This experiment was carried out to determine the nuclear localization of FOXO3.

Furthermore, PINK1 and Parkin co-localization staining was conducted to study the interaction between PINK1 and Parkin in mitophagy. Cells were incubated with rabbit anti-PINK1 (ab216144, 1:500, Abcam) and anti-Parkin (A0968, 1:100, Abclonal, Wuhan, China) antibodies at 37 °C for 60 min. Next, cells were incubated with FITC-conjugated goat anti-rabbit secondary antibody at 37 °C in the dark for 60 min. Cells were then stained with DAPI for 10 min and mounted with 20 μL of mounting medium for immediate observation under a fluorescence microscope (Yao et al. 2022).

### Establishment and treatment of mouse models

A total of 48 male BALB/c nude mice (aged 6–8 weeks, 18–25 g; Vital River Laboratory Animal Technology Co., Ltd., Beijing, China) were used in this study. The mice were housed in separate cages in a specific pathogen-free (SPF) animal laboratory with a 12-h light–dark cycle, 60–65% humidity, and a temperature of 22–25 °C, with free access to food and water. After one week of acclimatization and health observation, the experimental procedures were conducted. All experimental procedures were approved by the Institutional Animal Care and Use Committee (IACUC) at Zhujiang Hospital, Southern Medical University. The animals were cared for in accordance with the *Guide for the Care and Use of Laboratory Animals* and received appropriate housing and management under the supervision of experienced technicians.

Protocol 1: A total of 24 mice were randomly divided into four groups: sh-NC group (xenografted with EC cells with shRNA NC), sh-SIRT1 group (xenografted with EC cells with sh-SIRT1), oe-NC group (xenografted with EC cells with oe-NC), and oe-SIRT1 group (xenografted with EC cells with oe-SIRT1), with six mice per group. Each group received a subcutaneous injection of EC cells (1 × 10^7^ cells suspended in 100 μL PBS) into the back to establish a subcutaneous tumor model. From the 8th day post-injection, tumor width (W) and length (L) were measured every 4 days using a caliper to monitor tumor growth. Tumor volume (V) was calculated using the formula V = (W^2^ × L)/2. From the 28th day after injection, mice were treated daily with intraperitoneal injections of the synthetic progestin medroxyprogesterone acetate (MA, HY-B0469, MedChemExpress, USA) at a dose of 100 mg/kg. Tumor size was determined every 4 days using a caliper. After 16 days of continuous treatment, mice were euthanized, and tumors were dissected, photographed, and weighed (Gu et al. 2011).

Protocol 2: Each mouse was injected subcutaneously with oe-SIRT1-transduced cells (1 × 10^7^ cells suspended in 100 μL PBS) to establish a subcutaneous tumor model. When the tumor size reached approximately 100 mm^3^, the mice were randomly divided into four groups: PBS group (control), MA group (100 mg/kg MA), CQ group (25 mg/kg CQ), and CQ + MA group (100 mg/kg MA combined with 25 mg/kg CQ), with six mice per group. Daily intraperitoneal injections were administered, and tumor size was measured every 4 days to calculate tumor volume. After 28 days of continuous treatment, mice were euthanized, and tumors were excised, photographed, and weighed (Chen et al. 2024).

### TUNEL assay

Tumor tissue sections from mice were fixed in 4% paraformaldehyde for 15 min and permeabilized with 0.1% Triton-X 100 in PBS for 3 min. The TUNEL staining kit (C1090, Beyotime) was used to stain the cells. Cells were fixed again with 4% paraformaldehyde for 30 min and then incubated with PBS containing 0.3% Triton X-100 at room temperature for 5 min. Next, 50 μL of TUNEL detection solution was added, and the samples were incubated in the dark at 37 °C for 60 min. The samples were counterstained with DAPI (10 μg/mL) for 10 min and then sealed with an anti-fluorescence quenching mounting medium. Cy3 fluorescence (excitation at 550 nm, emission at 570 nm) was observed under a fluorescence microscope (Han et al. 2020). Image Pro Plus 6.0 software was used to calculate the apoptosis ratio.

### Immunohistochemical staining

Paraffin-embedded tissue sections were deparaffinized in xylene for 10 min twice and rehydrated in a graded ethanol series (100%, 95%, 70%) for 5–10 min each. Sections were then microwaved in 0.01 M citrate buffer (pH 6.0) for antigen retrieval, followed by cooling to room temperature. After washing with PBS three times for 3 min each, sections were incubated with 3% H_2_O_2_ at room temperature to inactivate endogenous peroxidase activity. Subsequently, sections were blocked with normal goat serum (E510009, Sangon Biotech) for 20 min at room temperature.

Sections were incubated with primary antibodies against SIRT1 (ab76039, 1:300), FOXO3 (ab314007, 1:180), LC3B (ab192890, 1:1000), and p62 (ab207305, 1:2000, Abcam) overnight at 4 °C. Sections were incubated with goat anti-mouse or anti-rabbit IgG secondary antibodies for 30 min, followed by incubation with SABC (P0603, Beyotime) at 37 °C for 30 min. DAB substrate was added for color development, followed by counterstaining with hematoxylin. Sections were dehydrated through graded ethanol series and cleared in xylene before being mounted with neutral resin. Observations were made under a brightfield microscope (BX63, Olympus, Japan) (Che et al. 2020).

### Tissue immunofluorescence

Tissue sections were fixed with 4% paraformaldehyde for 15–30 min. Subsequently, cells were permeabilized with 0.1% Triton for 15 min and blocked with PBS containing 15% FBS at 5 °C overnight.

FOXO3 nuclear localization staining was further conducted. Sections were incubated with rabbit anti-FOXO3 antibody at 37 °C for 60 min and then incubated with FITC-conjugated goat anti-rabbit secondary antibody at 37 °C for 60 min. Subsequently, tissues were stained with Alexa-488 conjugated goat anti-rabbit antibody and DAPI for 1 h at room temperature. Sections were mounted with 20 μL of mounting medium for observation under a fluorescence microscope (Wagle et al. 2016).

PINK1 and Parkin co-localization staining was adopted to visualize and quantify the localization of proteins and their interactions. Sections were incubated with rabbit anti-PINK1 and anti-Parkin antibodies at 37 °C for 60 min and then incubated with FITC-conjugated goat anti-rabbit secondary antibody at 37 °C for 60 min. Subsequently, sections were stained with DAPI for 10 min and mounted with 20 μL mounting medium for immediate observation under a fluorescence microscope (Yao et al. 2022).

### Statistical analysis

Data were derived from at least three independent experiments and are presented as mean ± standard deviation (SD). The independent samples t-test was employed for the comparison between two groups and the one-way analysis of variance (ANOVA) was used for comparisons among three or more groups. If ANOVA indicated significant differences, Tukey's HSD post-hoc test was performed to compare differences between groups. For non-normally distributed data or unequal variances, the Mann–Whitney U test or Kruskal–Wallis H test was applied. All statistical analyses were conducted using GraphPad Prism 9.5.0 (GraphPad Software, Inc.) and R version 4.2.1 (R Foundation for Statistical Computing). A significance level of 0.05 was set for all tests, with two-sided p values < 0.05 considered statistically significant.

## Results

### Mitophagy dysfunction in EC and characterization of key genes

**Fig. 1:**
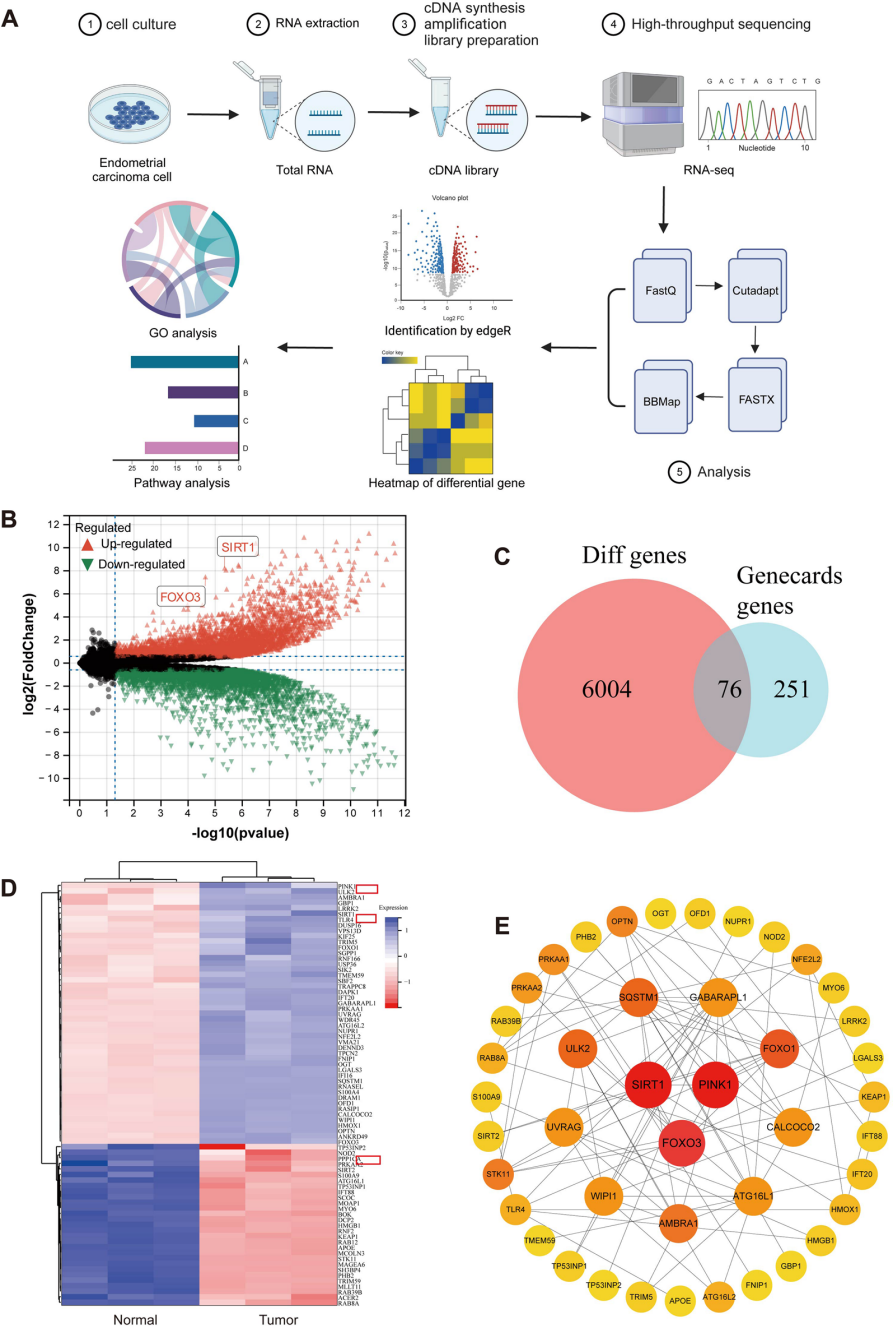
Transcriptional sequencing analysis of key genes associated with EC and mitophagy. **A** Volcano plot of gene expression in 3 groups of normal endometrial cells (EEC cells) and three groups of EC cells (RL95-2 cells). Upregulated genes are indicated by red triangles, downregulated genes by green triangles, and non-DEGs by black dots; **B** Venn diagram showing the intersection of DEGs and mitophagy-related genes; **C** Heatmap of DEGs intersecting between 3 groups of EEC cells and 3 groups of RL95-2 cells, with blue indicating upregulated genes and red indicating downregulated genes; **D** PPI network graph (Combined score = 0.7), with yellow to red color gradient indicating the degree values of the genes from small to large; **E** Bubble chart of the KEGG pathway enrichment analysis results for 76 EARGs; **F** Circle chart of the KEGG pathway enrichment analysis results for 76 EARGs

Initially, the differential expression of mitophagy-related genes was identified between normal endometrial cells (EEC) and EC cells (RL95-2). High-throughput transcriptome sequencing analysis was performed with three replicates for each cell type (Figure S2A). After quality control and filtering of the raw data, differential expression analysis identified 6,080 EC-related genes (ERGs) based on criteria of |log2FC|> 1 and *p* < 0.05, including 3,091 downregulated and 2,998 upregulated genes (Fig. 1A).

Furthermore, we searched the Genecards website for "Autophagy" and obtained 327 significant mitophagy-related genes. Then, we intersected these genes with the 6,080 ERGs and obtained 76 genes that were significantly associated with both EC and mitophagy (EC- and autophagy-related genes, EARGs) (Fig. 1B). The expression of these 76 EARGs in normal endometrial cells and EC cells is shown in Fig. 1C, with notable upregulation of genes such as FOXO3, SIRT1, and PINK1 in EC cells.

To identify key protein interaction networks, STRING database analysis and Cytoscape 3.5.1 visualization with the CytoHubba tool highlighted central proteins such as SIRT1, FOXO3, PINK1, SQSTM1 (p62), and FOXO1, with SIRT1, FOXO3, and PINK1 being the most prominent (Fig. 1D, Table S4). Kaplan–Meier survival analysis of TCGA-UCEC patients, divided into high and low expression groups for each key gene, showed that high FOXO3 expression was associated with poor survival outcomes, while SIRT1 and PINK1 expressions did not significantly affect survival (Figure S2B).

GO enrichment analysis of the 76 EARGs indicated significant involvement in BPs such as regulation of autophagy, with CCs enriched in autophagosome and vacuolar membrane, and MFs in protein serine/threonine kinase activity and GTPase binding (Figure S2C-D). KEGG pathway enrichment highlighted the involvement in signaling pathways such as FOXO signaling pathway and autophagy-animal pathways (Fig. 1E, F), with SIRT1, FOXO3, and BNIP3 being key players in mitophagy (Figure S2E).

In this study, RNA-seq analysis of EC and normal endometrial cells revealed significant upregulation of SIRT1, FOXO3, and PINK1 in EC cells. These findings suggest that SIRT1, FOXO3, PINK1, and BNIP3 are involved in regulating mitophagy in EC cells, providing insights for potential therapeutic strategies.

### SIRT1 regulates FOXO3 protein activity in EC

**Fig. 2:**
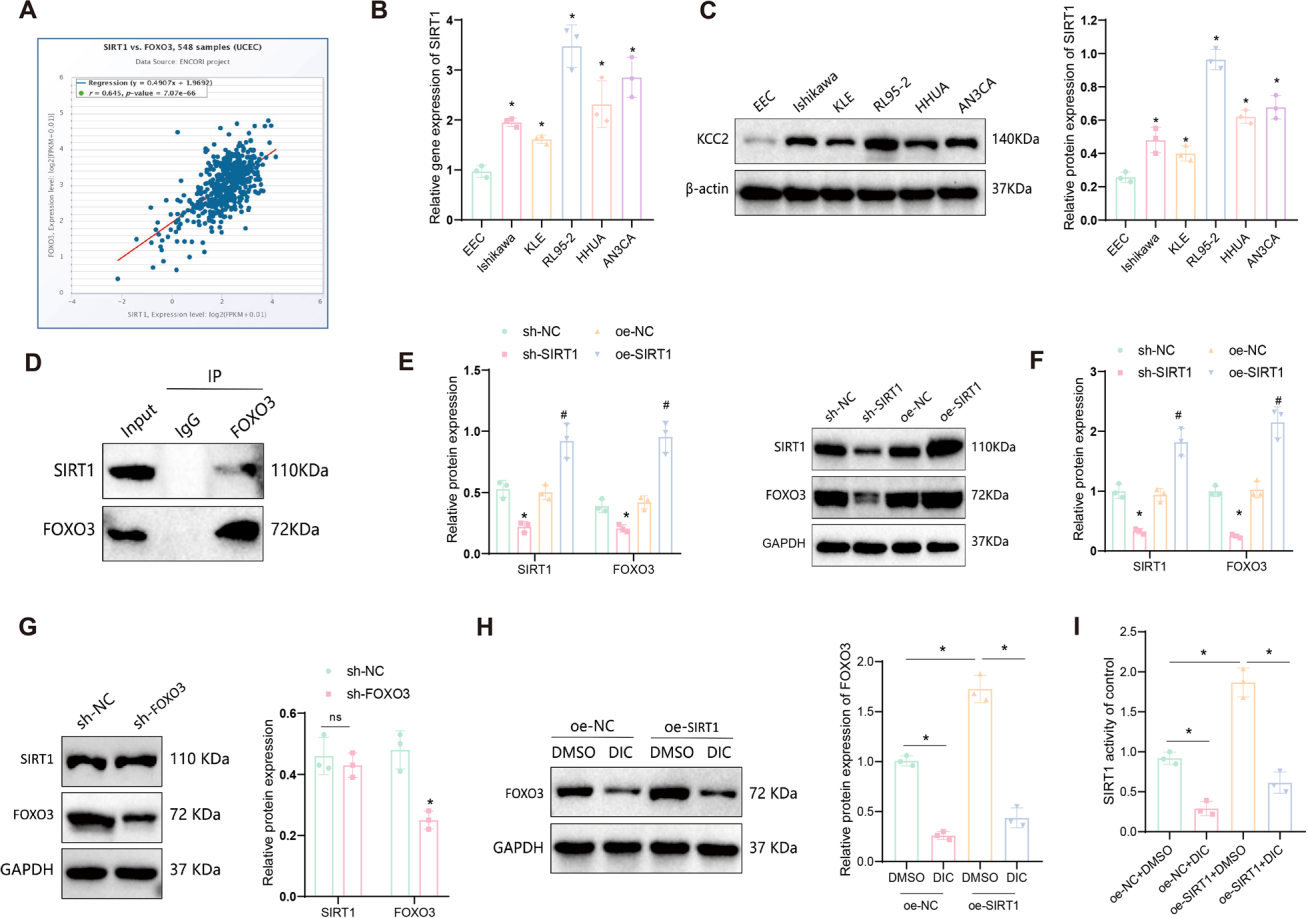
Regulation relationship between SIRT1 and FOXO3 proteins. **A** Correlation of SIRT1 and FOXO3 mRNA expression in EC (UCEC) according to starBase or ENCORI database, where r > 0 represents a positive correlation. **B** Detection of SIRT1 mRNA expression in different cell lines using RT-qPCR. **C** Detection of SIRT1 protein expression in different cell lines using Western blot; **D** Co-IP experiment to determine the interaction between SIRT1 and FOXO3 proteins in RL95-2 cells. **E** Detection of changes in SIRT1 and FOXO3 protein expression in RL95-2 cells after silencing or overexpressing SIRT1 mRNA using Western blot; **F** Detection of changes in SIRT1 and FOXO3 mRNA expression in RL95-2 cells after silencing or overexpressing SIRT1 mRNA using RT-qPCR; **G** Detection of changes in SIRT1 and FOXO3 protein expression in RL95-2 cells after silencing FOXO3 mRNA using Western blot; **H** Detection of the effect of overexpressing SIRT1 in RL95-2 cells and using the DIC on FOXO3 protein expression using Western blot; **I** Detection of the effect of the DIC treatment on SIRT1 activity. Data are presented as mean ± SD, and each cell experiment was repeated three times. *p < 0.05 compared to the EEC group or sh-NC group or between two groups; #*p* < 0.05 compared to the oe-NC group; ns indicates not significant

Previous research has reported that SIRT1 directly interacts with FOXO3 and regulates FOXO3 through deacetylation modification (Das et al. 2014). To investigate the interaction between SIRT1 and FOXO3 in EC cells, we examined the correlation between SIRT1 and FOXO3 mRNA expression in EC (UCEC) using the starBase or ENCORI database (https://rnasysu.com/encori/index.php). The results showed a positive correlation (r > 0) between SIRT1 and FOXO3 expression (Fig. 2A). Therefore, we hypothesized that SIRT1 might regulate the expression of FOXO3 in EC cells.

To validate this hypothesis, we first used RT-qPCR and Western blot to detect the expression of SIRT1 mRNA and protein in endometrial cells (EEC) and EC cell lines (Ishikawa, KLE, RL95-2, HHUA, and AN3CA). The results showed a significant increase in SIRT1 mRNA and protein expression in all EC cell lines compared to EEC cells, with RL95-2 cells exhibiting the highest expression (Fig. 2B, C). Therefore, we selected RL95-2 cells for subsequent experimental validation.

Additionally, we conducted Co-IP experiments in RL95-2 cells to confirm the interaction between SIRT1 and FOXO3 proteins, which was successfully validated (Fig. 2D). Subsequently, we either silenced or overexpressed SIRT1 in RL95-2 cells and validated the efficiency of SIRT1 silencing using RT-qPCR and Western blot. The most efficient cell lines were chosen for further analysis (Figure S3A-B). Silencing of SIRT1 in RL95-2 cells resulted in a significant decrease in FOXO3 mRNA and protein expression compared to the sh-NC group. Conversely, overexpressing SIRT1 led to a significant increase in FOXO3 mRNA and protein expression compared to the oe-NC group (Fig. 2E, F). Additionally, we silenced FOXO3 in RL95-2 cells and confirmed the efficiency (Figure S3C, D). However, silencing FOXO3 did not significantly affect SIRT1 protein levels (Fig. 2G).

To further confirm that SIRT1 regulates FOXO3 expression through deacetylation, RL95-2 cells were treated with a DIC. Western blot analysis showed that overexpression of SIRT1 increased FOXO3 protein levels, while treatment with DIC in both oe-NC and oe-SIRT1 groups effectively reduced FOXO3 protein stability compared to the DMSO controls (Fig. 2H). Moreover, DIC significantly inhibited SIRT1 protein activity (Fig. 2I). These results demonstrate that SIRT1 regulates FOXO3 protein expression through deacetylation in EC cells.

### SIRT1 regulates malignant phenotypes in EC cells

**Fig. 3:**
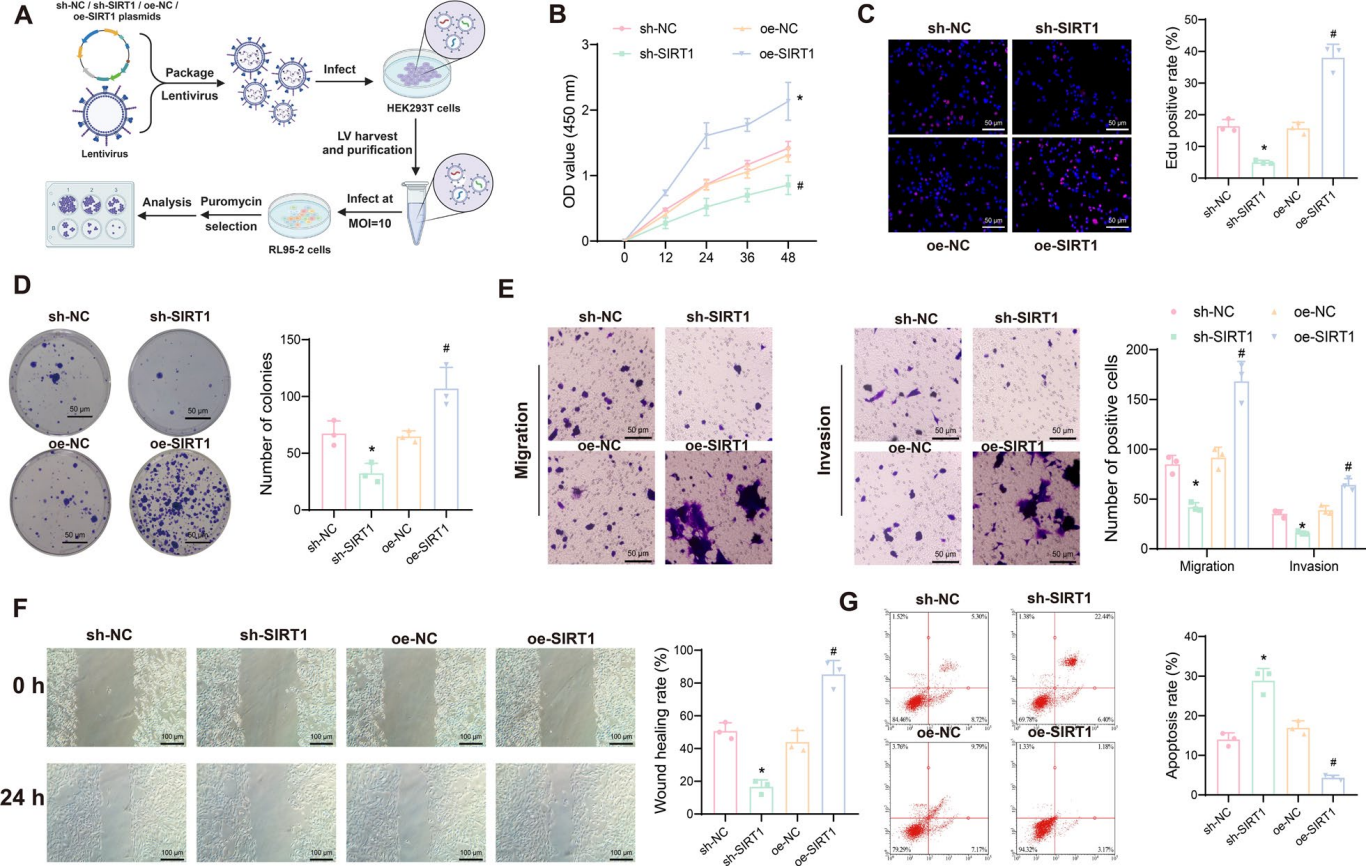
Effects of silencing or overexpressing SIRT1 on EC cell growth, proliferation, migration, and invasion. **A** Schematic diagram of the cell experiments; **B** Viability changes of RL95-2 cells after silencing or overexpressing SIRT1 at 12, 24, 36, and 48 h measured using the CCK-8 assay; **C** Proliferation capacity of RL95-2 cells after silencing or overexpressing SIRT1 detected using the EdU assay, where EdU-positive cells appear pink, and EdU-negative cells appear blue; **D** Colony formation assay to measure the colony formation ability of RL95-2 cells after silencing or overexpressing SIRT1; **E** Transwell assay to evaluate the migration and invasion capacity of RL95-2 cells after silencing or overexpressing SIRT1; **F** Wound healing assay to assess the migration of RL95-2 cells after silencing or overexpressing SIRT1; **G** Flow cytometry analysis to detect apoptosis of RL95-2 cells after silencing or overexpressing SIRT1. Data are presented as mean ± SD, and each cell experiment was repeated three times. *p < 0.05 compared to the sh-NC group; ^#^*p* < 0.05 compared to the oe-NC group

SIRT1 is a critical NAD-dependent deacetylase that plays a key role in various BPs, including maintaining mitochondrial function, promoting mitochondrial biogenesis, and regulating the autophagy-lysosome pathway. These functions contribute to cell growth, proliferation, migration, and invasion (Ou et al. 2014). To investigate the regulatory role of SIRT1 in the growth and proliferation of EC cells, we constructed SIRT1-silenced and SIRT1-overexpressing RL95-2 cell lines. The changes in cell growth, proliferation, migration, and invasion in different intervention groups were assessed using CCK-8, EdU staining, colony formation assays, Transwell assays, wound healing assays, and flow cytometry (Fig. 3A).

The results indicated that compared to the sh-NC group, SIRT1 silencing (sh-SIRT1) significantly reduced cell viability and proliferation (Fig. 3B, C), colony formation ability (Fig. 3D), and migration and invasion abilities (Fig. 3E, F), while increasing cell apoptosis (Fig. 3G). Conversely, overexpression of SIRT1 enhanced cell proliferation, colony formation, migration, and invasion, and inhibited apoptosis.

These findings demonstrate that silencing SIRT1 in EC cell lines inhibits cell growth, proliferation, migration, and invasion while promoting apoptosis. In contrast, overexpressing SIRT1 promotes these processes and inhibits apoptosis, highlighting the critical role of SIRT1 in regulating the fate of EC cells.

### SIRT1 regulates mitophagy and cell fate in EC cells

**Fig. 4:**
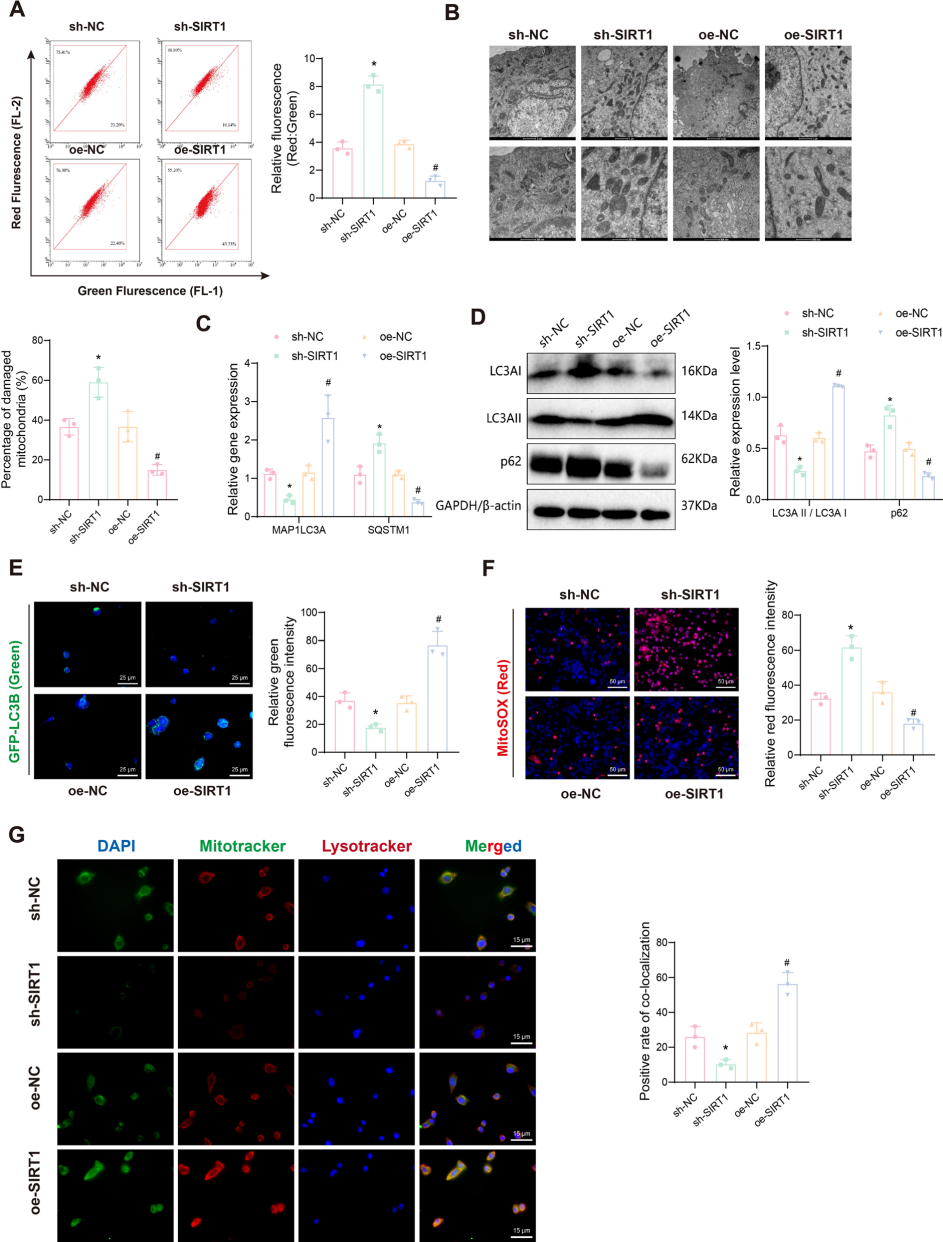
Impact of SIRT1 silencing or overexpression on mitophagy and mitochondrial function in EC cells. **A** JC-1 staining experiment to assess the change in MMP in RL95-2 cells after silencing or overexpressing SIRT1; **B** TEM to examine mitochondrial ultrastructure; **C** RT-qPCR to measure the expression changes of MAP1LC3A and SQSTM1 mRNA after silencing or overexpressing SIRT1; **D** Western blot analysis to detect the expression changes of LC3AI, LC3AII, and p62 proteins after silencing or overexpressing SIRT1 (note: LC3AI and LC3AII bands are displayed on the same gel image); **E** Immunofluorescence staining to assess the transfection efficiency of GFP-LC3B plasmid after silencing or overexpressing SIRT1; **F** MitoSOX immunofluorescence staining to examine the regulation of cellular ROS generation in response to silencing or overexpression of SIRT1; **G** Immunofluorescence co-localization staining of Mitotracker and Lysotracker to investigate the regulation of cellular mitophagy in response to silencing or overexpression of SIRT1. Data are presented as mean ± SD, with each cellular experiment repeated 3 times. *p < 0.05 compared to the sh-NC group; ^#^*p* < 0.05 compared to the oe-NC group

To investigate whether SIRT1 regulates mitophagy in EC cells, JC-1 staining was used to detect changes in MMP across different intervention groups. The results indicated that, compared to the sh-NC group, the sh-SIRT1 group showed reduced green fluorescence and increased red fluorescence, with a higher red/green fluorescence ratio, indicating mitochondrial depolarization. Conversely, the oe-SIRT1 group showed a lower red/green fluorescence ratio compared to the oe-NC group (Fig. 4A). Next, we examined mitochondrial damage in EC cells using TEM. The results demonstrated that SIRT1 knockdown increased mitochondrial damage, whereas SIRT1 overexpression reduced it (Fig. 4B).

LC3 is an autophagosome marker critical for its formation, and p62 links LC3 to ubiquitinated proteins, with its expression negatively correlating with autophagy activity (Klionsky et al. 2016). RT-qPCR results showed that, compared to the sh-NC group, the sh-SIRT1 group had significantly lower MAP1LC3A mRNA (encoding LC3A) and higher SQSTM1 mRNA (encoding p62) levels. Overexpression of SIRT1 led to increased MAP1LC3A mRNA and decreased SQSTM1 mRNA levels (Fig. 4C). Western blot results indicated that SIRT1 knockdown reduced LC3AII expression, increased LC3AI expression, and lowered the LC3AII/LC3AI ratio while increasing p62 levels. Conversely, overexpressing SIRT1 raised the LC3AII/LC3AI ratio and decreased p62 expression (Fig. 4D), suggesting that SIRT1 promotes autophagy in EC cells.

Immunofluorescence staining of GFP-LC3B plasmid transfection showed that green fluorescence decreased in the sh-SIRT1 group compared to the sh-NC group, while it increased in the oe-SIRT1 group compared to the oe-NC group (Fig. 4E). MitoSOX staining revealed that SIRT1 knockdown significantly increased ROS production (enhanced red fluorescence) and promoted apoptosis, whereas SIRT1 overexpression slightly increased ROS production and promoted autophagy (Fig. 4F). Furthermore, co-localization analysis with Mitotracker (mitochondrial marker) and Lysotracker (autophagosome marker) indicated reduced co-localization in the sh-SIRT1 group and increased co-localization in the oe-SIRT1 group (Fig. 4G).

To further validate that SIRT1 overexpression promotes cell growth, proliferation, migration, and invasion by enhancing mitophagy and inhibiting apoptosis, we treated oe-SIRT1 cells with the autophagy inhibitor CQ (Kimura et al. 2013). CQ treatment reduced cell viability and proliferation (Figure S4A-B), decreased colony formation (Figure S4C), and weakened proliferation and migration abilities in EC cells (Figure S4D). CQ significantly promoted apoptosis and increased MMP (Figure S4E-F). TEM results showed that CQ promoted mitochondrial damage in oe-SIRT1 cells (Figure S4G). Immunofluorescence results indicated that CQ significantly increased ROS production and inhibited GFP-LC3B expression in oe-SIRT1 cells (Figure S4H-I). CQ treatment reversed the effects of SIRT1 overexpression on LC3AII, LC3AI, and p62 expression in EC cells (Figure S4J-K). These findings confirm that SIRT1 promotes mitophagy by deacetylating FOXO3, thereby enhancing cell growth, proliferation, migration, and invasion.

The results further verify that SIRT1 knockdown inhibits mitophagy in EC cells, while SIRT1 overexpression promotes it. Overall, these findings preliminarily confirm that SIRT1 overexpression promotes cell growth, proliferation, migration, and invasion by enhancing mitophagy and inhibiting apoptosis in EC cells.

### SIRT1 induces mitophagy in EC cells through FOXO3 deacetylation

**Fig. 5:**
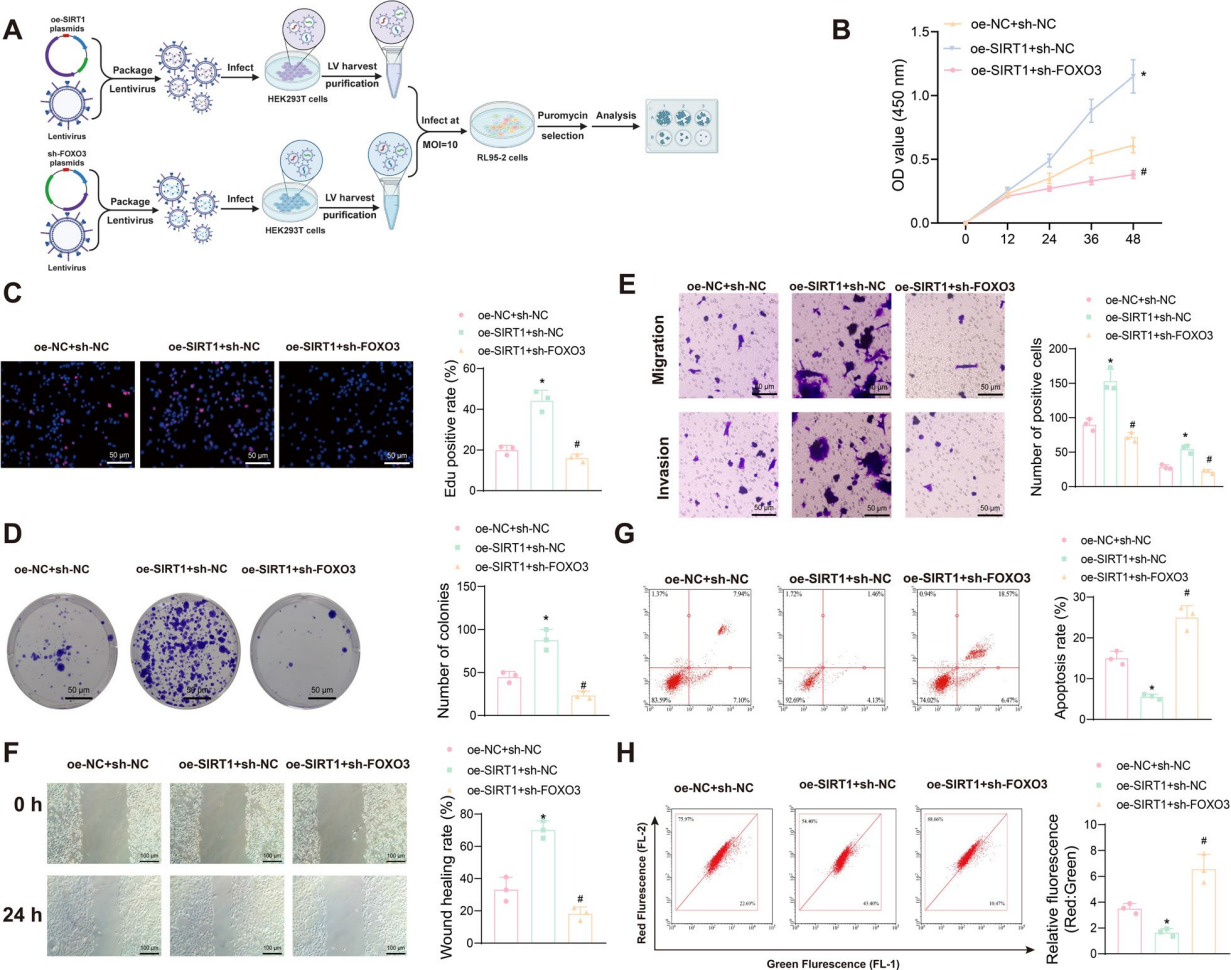
Impact of SIRT1 overexpression on EC cell survival via FOXO3. **A** Schematic diagram of the experimental procedure; **B** CCK-8 assay to measure the viability changes of EC cells in different intervention groups at 12, 24, 36, and 48 h; **C** EdU assay to assess the proliferation ability of EC cells in different intervention groups, with EdU-positive cells shown in pink and EdU-negative cells shown in blue; **D** Colony formation assay to evaluate the colony-forming ability of EC cells in different intervention groups; **E** Transwell assay to investigate the migration and invasion ability of EC cells in different intervention groups; **F** Wound healing assay to examine the migration of EC cells in different intervention groups; **G** Flow cytometry analysis to detect the apoptosis of EC cells in different intervention groups; **H** JC-1 staining experiment to assess the change in MMP of EC cells in different intervention groups. Data are presented as mean ± SD, with each cellular experiment repeated 3 times. *p < 0.05 compared to the oe-NC + sh-NC group; ^#^*p* < 0.05 compared to the oe-SIRT1 + sh-NC group

To further verify the role of SIRT1 in inducing mitophagy through deacetylation of FOXO3 in EC cells, we overexpressed SIRT1 in RL95-2 cells and subsequently knocked down FOXO3 (Fig. 5A). EdU assay results indicated that FOXO3 knockdown reversed the promotive effects of SIRT1 overexpression on cell viability and proliferation in EC cells (Fig. 5B, C). Additionally, FOXO3 knockdown significantly reduced SIRT1 overexpression-driven colony formation (Fig. 5D), proliferation, and migration capabilities (Fig. 5E, F), and increased SIRT1 overexpression-inhibited apoptosis (Fig. 5G) and MMP (Fig. 5H) in EC cells. These results suggest that FOXO3 knockdown can counteract the promotive effects of SIRT1 overexpression on EC cell viability, proliferation, migration, and invasion, and the inhibitory effects on apoptosis.

**Fig. 6:**
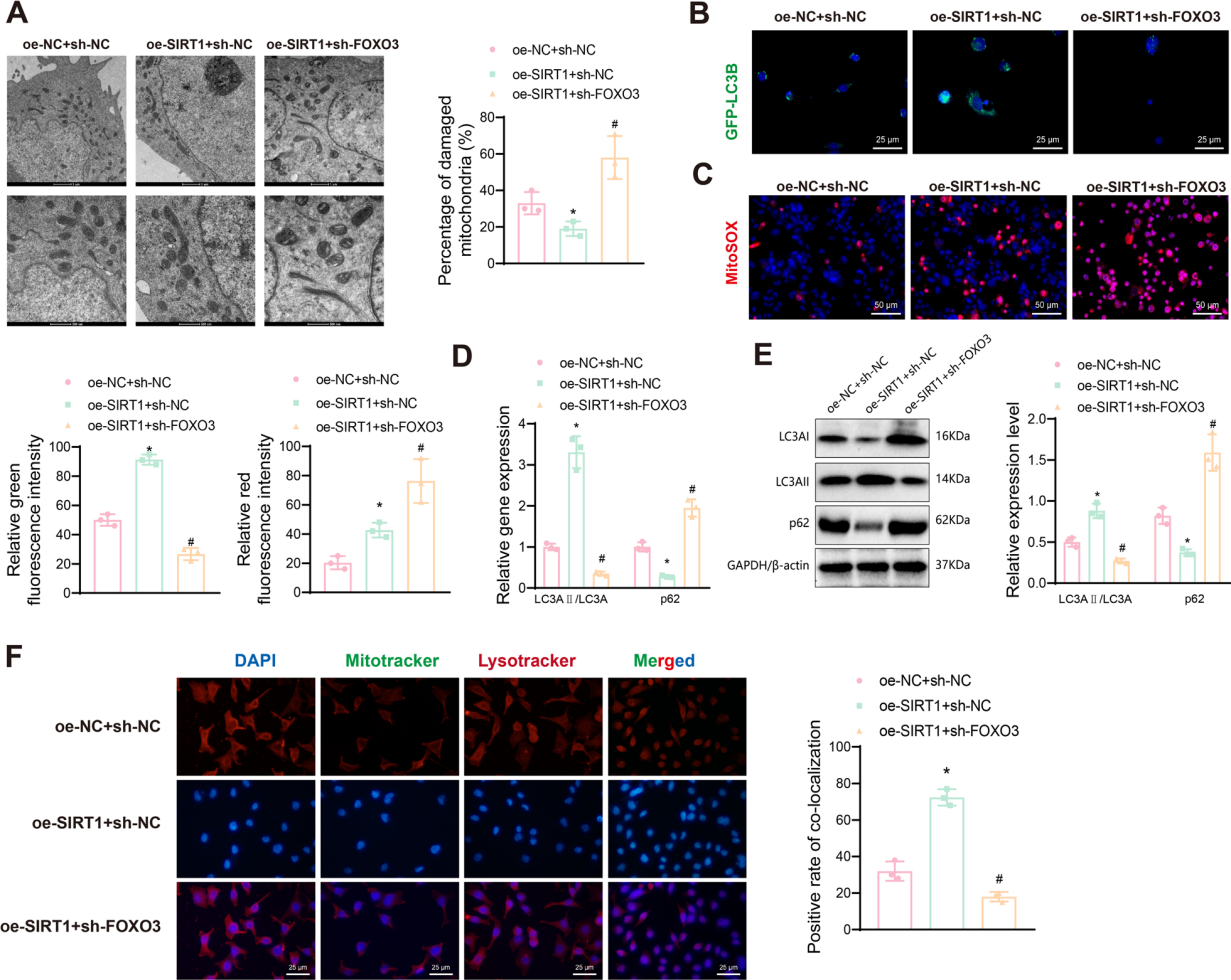
Effects of SIRT1 overexpression on FOXO3-mediated mitophagy in EC cells. **A** TEM examines mitochondrial ultrastructure; **B** Immunofluorescence staining examines transfection of GFP-LC3B plasmid in different intervention groups of EC cells; **C** MitoSOX immunofluorescence staining detects variations in ROS generation in different intervention groups of EC cells; **D** RT-qPCR detects changes in MAP1LC3A and SQSTM1 mRNA expression in different intervention groups of EC cells; **E** Western blot detects changes in LC3AI, LC3AII, and p62 protein expression in different intervention groups of EC cells (note: LC3AI and LC3AII bands should be presented on one gel image); **F** Mitotracker and Lysotracker immunofluorescence co-staining determines the co-localization of mitochondria and autophagosomes in different intervention groups of EC cells. Data are presented as mean ± SD, and each cell experiment in each group was repeated 3 times. *p < 0.05 compared to oe-NC + sh-NC group, with *p < 0.05; ^#^*p* < 0.05 compared to oe-SIRT1 + sh-NC group, with ^#^*p* < 0.05

Further analysis revealed that SIRT1 overexpression promotes EC cell proliferation, migration, and invasion while inhibiting apoptosis, and that FOXO3 silencing can reverse these effects. To verify that SIRT1 regulates FOXO3 to mediate mitophagy, TEM experiments showed that FOXO3 knockdown reversed the protective effects of SIRT1 overexpression on mitochondrial damage (Fig. 6A). Immunofluorescence staining demonstrated that FOXO3 knockdown reversed the SIRT1 overexpression-induced increase in GFP-LC3B expression (Fig. 6B). MitoSOX staining revealed that FOXO3 knockdown in SIRT1-overexpressing cells significantly increased ROS production and promoted apoptosis (Fig. 6C). RT-qPCR and Western blot analyses indicated that FOXO3 knockdown reversed the regulatory effects of SIRT1 overexpression on LC3AII, LC3AI, and p62 expression in EC cells (Fig. 6D, E). In addition, Mitotracker and Lysotracker co-localization experiments (Fig. 6F) demonstrated that FOXO3 knockdown decreased the co-localization of mitochondria and autophagosomes in SIRT1-overexpressing EC cells, thereby reducing mitophagy.

These findings further confirm that SIRT1 promotes mitophagy in EC cells through the deacetylation of FOXO3, which in turn enhances cell growth, proliferation, migration, and invasion while inhibiting apoptosis.

### SIRT1 enhances mitophagy in EC cells through FOXO3 and PINK1/parkin pathways

**Fig. 7:**
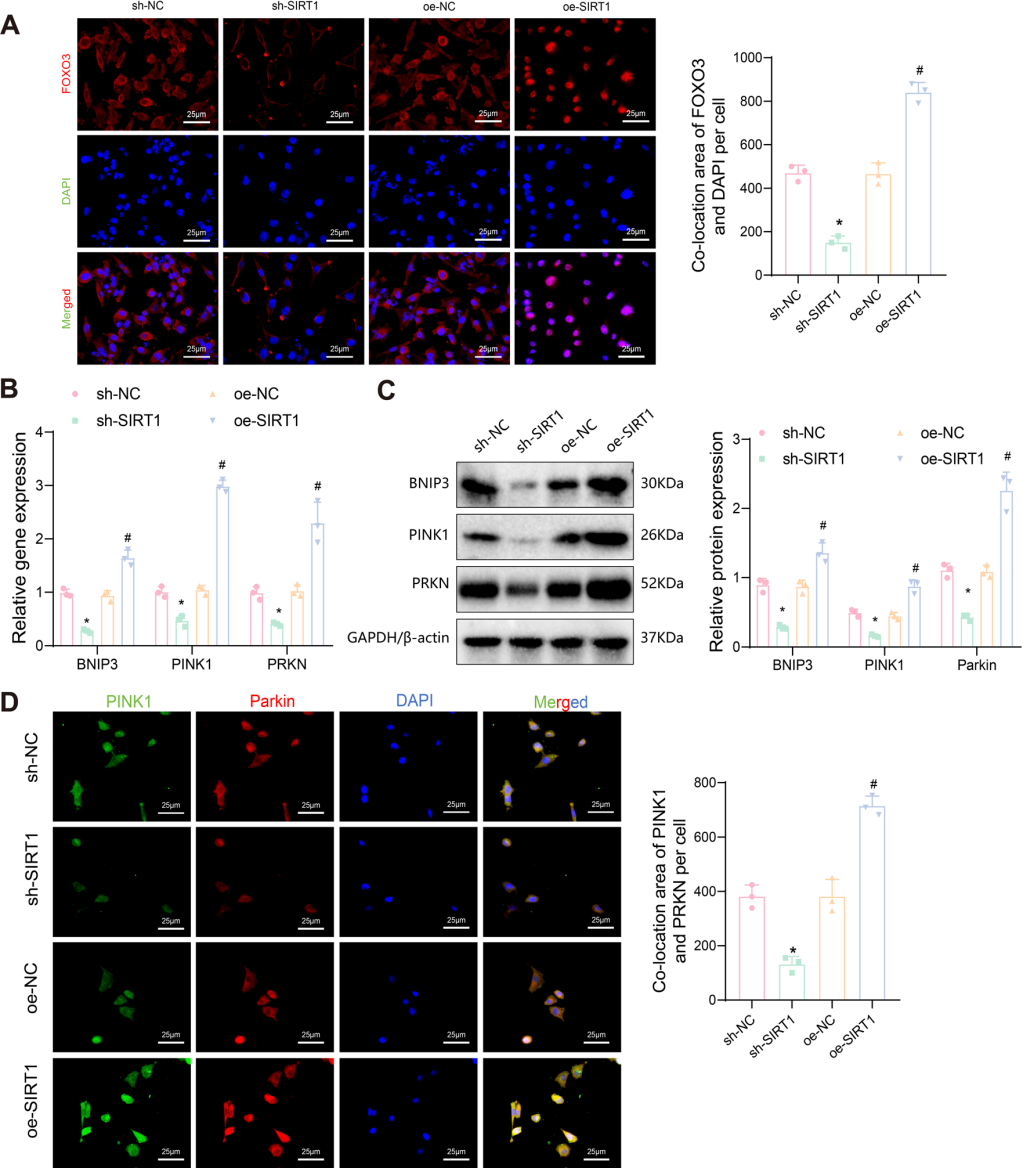
Effects of SIRT1 overexpression on BNIP3/PINK1/Parkin-mediated mitophagy in EC cells. **A** Immunofluorescence staining determines the subcellular localization of FOXO3 protein in different intervention groups of EC cells; **B** RT-qPCR detects the expression of BNIP3, PINK1, and PRKN mRNA in different intervention groups of EC cells; **C** Western blot detects the expression of BNIP3, PINK1, and Parkin protein in different intervention groups of EC cells; **D** Immunofluorescence staining examines the expression and co-localization of PINK1 and Parkin protein in different intervention groups of EC cells. Data are presented as mean ± SD, and each cell experiment in each group was repeated 3 times. *p < 0.05 compared to sh-NC group, with *p < 0.05; ^#^*p* < 0.05 compared to oe-NC group, with #*p* < 0.05

Previous experiments demonstrated that SIRT1 overexpression in EC cells can promote FOXO3 protein deacetylation, upregulate FOXO3 protein expression, and enhance mitophagy, thereby promoting cell growth, proliferation, migration, and invasion. High-throughput transcriptome sequencing and bioinformatics analysis revealed significant upregulation of the FOXO signaling pathway in EC cells. To further investigate the specific pathways by which SIRT1/FOXO3 regulates mitophagy, we first examined the nuclear localization of the FOXO3 protein using immunofluorescence. The results showed that silencing SIRT1 inhibited FOXO3 expression and its nuclear translocation, while overexpressing SIRT1 promoted FOXO3 expression and its nuclear translocation (Fig. 7A).

FOXO3 is known to regulate mitochondrial function and integrity by binding to the upstream promoter region of BNIP3, thereby increasing BNIP3 expression (Lu et al. 2014). BNIP3, a member of the Bcl-2 family, functions as a mitophagy receptor and induces MMP depolarization, triggering mitophagy (Zhang and Ney 2009; Hu et al. 2016). Moreover, the SIRT1/FOXO3/BNIP3 axis has been identified as an important regulatory signaling pathway of the PINK1/Parkin ubiquitin-conjugating system, which promotes PINK1-mediated mitophagy (Yao et al. 2022). PINK1 accumulates on the outer mitochondrial membrane in response to mitochondrial depolarization and phosphorylates, recruiting Parkin to initiate mitophagy (Li et al. 2022; Huang and Mu 2016).

We first assessed the expression of BNIP3, PINK1, and PRKN in different intervention groups using RT-qPCR and Western blot. The results showed that, compared to the sh-NC group, BNIP3, PINK1, and PRKN mRNA and their encoded proteins were significantly downregulated in the sh-SIRT1 group. Conversely, these expressions were significantly upregulated in the oe-SIRT1 group compared to the oe-NC group (Fig. 7B, C). Furthermore, immunofluorescence staining was used to detect the localization of PINK1 and Parkin proteins. The results confirmed that silencing SIRT1 inhibited the expression of PINK1 and Parkin proteins and their co-localization, while overexpressing SIRT1 enhanced their expression and co-localization (Fig. 7D).

Taken together, these findings suggest that SIRT1 overexpression in EC cells promotes FOXO3 protein deacetylation, enhancing FOXO3 expression, which in turn may upregulate BNIP3 transcription and promote mitophagy through the PINK1/Parkin pathway.

### SIRT1 Modulates Tumor Growth and Hormone Resistance in EC Cells

Previous studies have reported that overexpression of SIRT1 significantly enhances resistance to cisplatin and paclitaxel in vitro, and overexpression of SIRT1 accelerates the growth of tumor xenografts in nude mice and cisplatin resistance in vivo (Asaka et al. 2015). Increased activity of SIRT1 leads to upregulation of resistance-promoting genes in drug-resistant cancer cell lines, whereas inhibition of SIRT1 with siRNA reduces cell resistance (Moore et al. 2012; Chu et al. 2005). Furthermore, SIRT1 is upregulated in hormone-resistant cells and is significantly associated with hormone resistance (Wang et al. 2018). These results suggest that SIRT1 may be involved in the transition of cancer cells from drug-responsive to drug-resistant phenotypes, making SIRT1 activity a potential prognostic indicator for chemotherapy response.

**Fig. 8:**
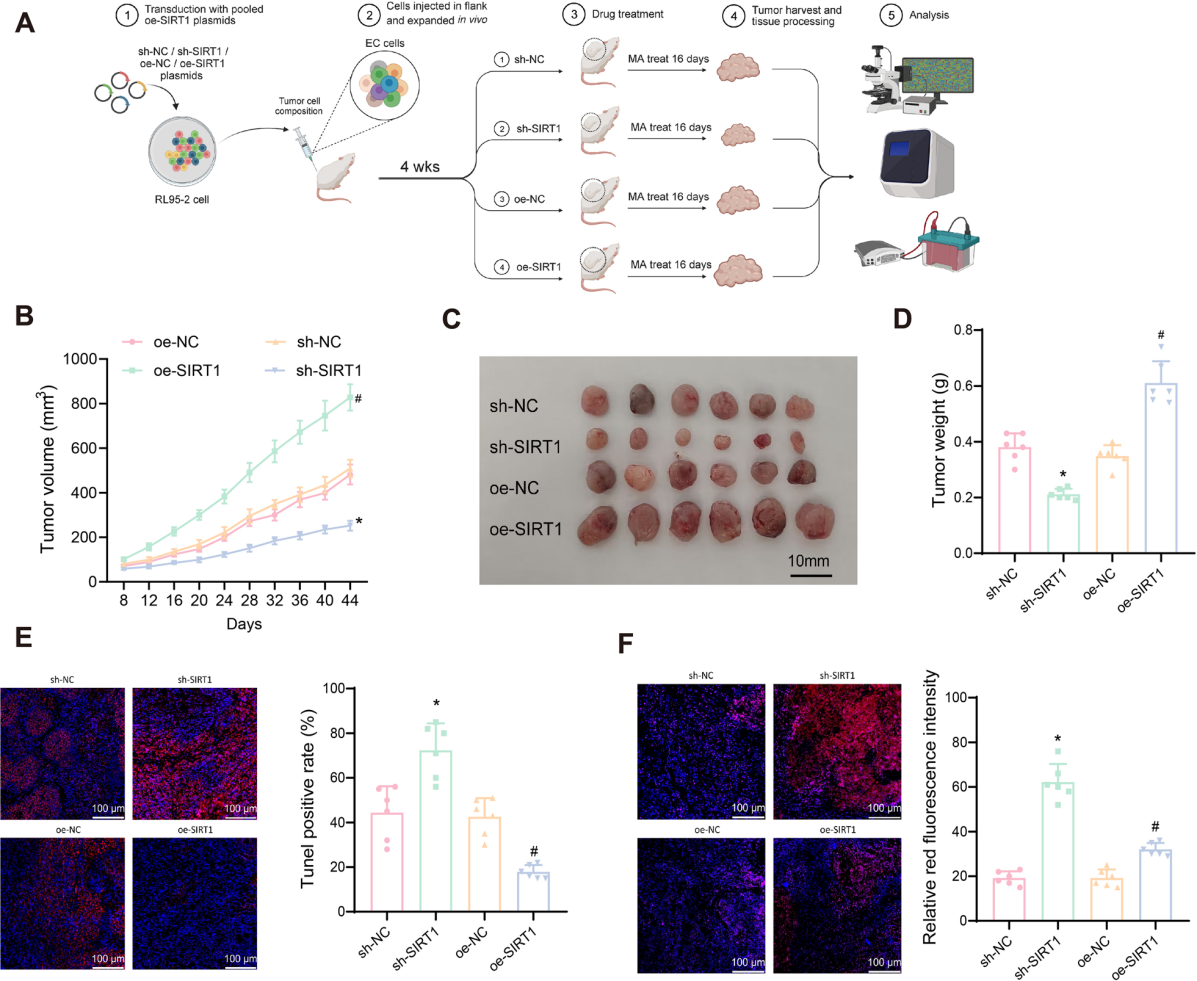
Overexpression of SIRT1 enhances the intracellular growth of EC cells. **A** Diagram illustrating the procedure of animal experiments (n = 6); **B** Line graph showing the tumor volume increase in subcutaneous tumor model mice from day 8 to 44 (n = 6); **C** Dissection of subcutaneous transplanted tumors in mice from each group on day 44 (n = 6); **D** Statistical analysis of tumor weight in subcutaneous tumor model mice on day 44 (n = 6); **E** TUNEL staining for apoptosis in mouse tumors from each group (n = 6); **F** MitoSOX immunofluorescence staining for ROS production in mouse tumor tissues from each group (n = 6). Data are presented as mean mean ± SD, with 6 nude mice in each group. *p < 0.05 compared to sh-NC group; #*p* < 0.05 compared to oe-NC group

To explore whether SIRT1 deacetylation of FOXO3 regulating mitophagy induces hormone resistance in EC, we constructed SIRT1-silenced and SIRT1-overexpressing cell xenograft mouse models. Tumor volumes were measured every 4 days starting from day 8 post-implantation (Fig. 8A). After 28 days, the average tumor volume in the oe-SIRT1 group increased to (1381.01 ± 77.53) mm^3^, which was significantly larger than that in the control oe-NC group (985.17 ± 75.21) mm^3^. In contrast, the tumor volume in the sh-SIRT1 group was (374.17 ± 39.83) mm^3^, much smaller than that in the oe-SIRT1 control group (1005.66 ± 108.05) mm^3^ (Fig. 8B). On the 28th day of tumor injection, mice were treated with the synthetic progestin MA at a concentration of 100 mg/kg for 16 consecutive days. Tumor growth slowed in the sh-NC and oe-NC groups after drug administration, with the sh-SIRT1 group tumors gradually shrinking, while the oe-SIRT1 group tumors continued to grow rapidly (Fig. 8B).

After 16 days of continuous treatment, tumors were dissected, photographed, and weighed. The results showed that, compared to the sh-NC group, the tumor volume and weight in the sh-SIRT1 group were significantly reduced, while in the oe-SIRT1 group, the tumor volume and weight were significantly increased compared to the oe-NC group (Fig. 8C, D). Tumor weight in the sh-SIRT1 group decreased by approximately 60% compared to the sh-NC group, while tumor weight in the oe-SIRT1 group increased by about 50% compared to the oe-NC group (Fig. 8D).

Furthermore, TUNEL assays were used to detect apoptosis in tumor cells from each group. Results showed a significant increase in apoptosis in the sh-SIRT1 group compared to the sh-NC group, while apoptosis was significantly reduced in the oe-SIRT1 group compared to the oe-NC group (Fig. 8E). MitoSOX immunofluorescence staining indicated that SIRT1 silencing in EC cells significantly increased ROS production and promoted apoptosis, whereas SIRT1 overexpression slightly increased ROS production and promoted autophagy (Fig. 8F).

These findings indicate that SIRT1 overexpression in EC cell lines promotes tumor growth and hormone resistance in vivo, while SIRT1 silencing inhibits tumor growth and increases sensitivity to hormone treatment.

### *SIRT1 Enhances Hormone Resistance *via* mitophagy*

**Fig. 9:**
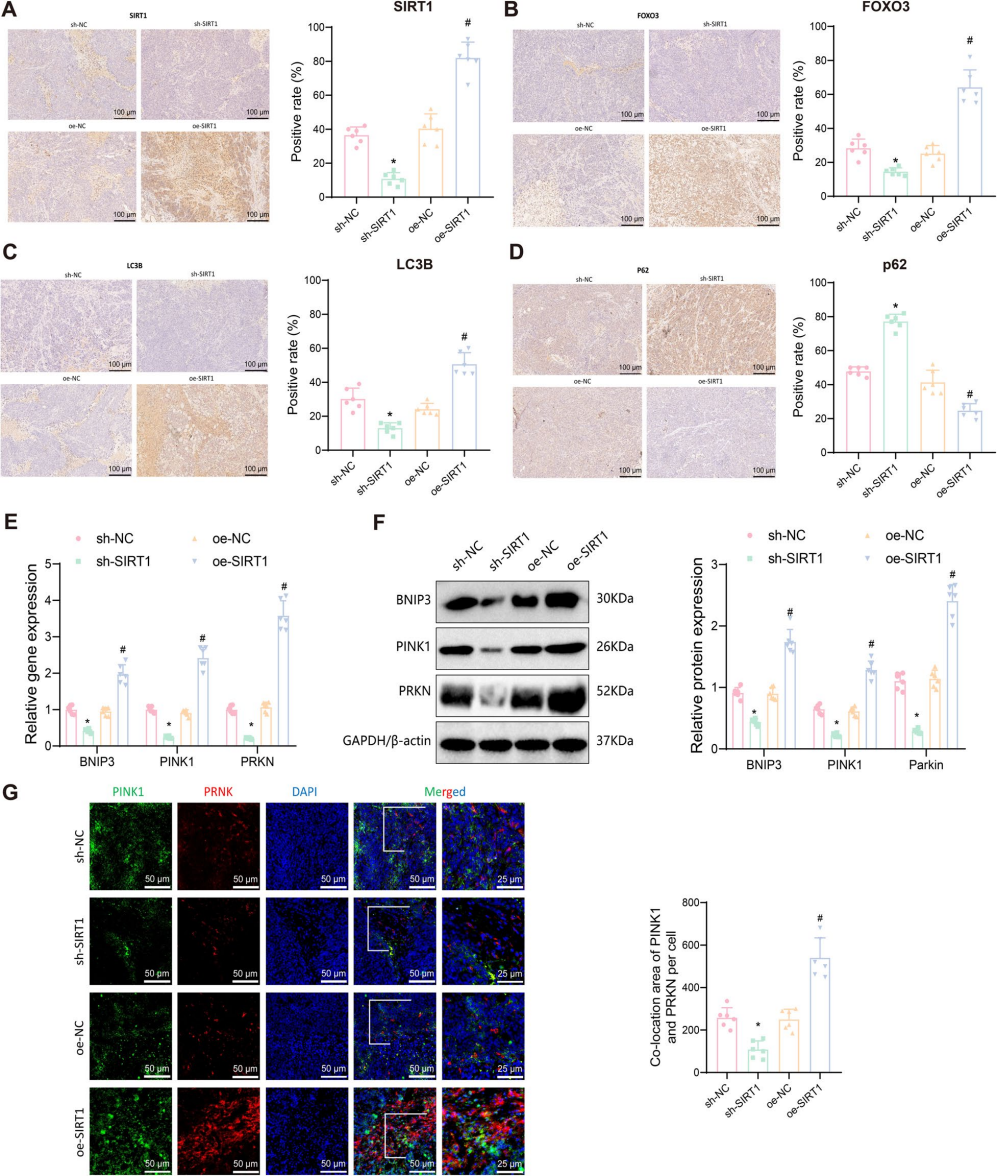
Overexpression of SIRT1 enhances tumor growth through the BNIP3/PINK1/Parkin pathway. **A** Immunohistochemical staining showing the expression changes of SIRT1 protein in mouse tumor tissues from each group (n = 6); **B** Immunohistochemical staining showing the expression changes of FOXO3 protein in mouse tumor tissues from each group (n = 6); **C** Immunohistochemical staining showing the expression changes of LC3B protein in mouse tumor tissues from each group (n = 6); **D** Immunohistochemical staining showing the expression changes of p62 protein in mouse tumor tissues from each group (n = 6); **E** RT-qPCR analysis of the expression of BNIP3, PINK1, and PRKN mRNA in mouse tumor tissues from each group; **F** Western blot analysis of the expression of BNIP3, PINK1, and Parkin proteins in mouse tumor tissues from each group; **G** Immunofluorescence staining showing the expression and co-localization of PINK1 and Parkin proteins in mouse tumor tissues from each group. Data are presented as mean mean ± SD. *p < 0.05 compared to sh-NC group; ^#^*p* < 0.05 compared to oe-NC group

To further explore the mechanism by which SIRT1 induces hormone resistance, we examined the expression of SIRT1, FOXO3, LC3B, and p62 proteins in EC tumor tissues from mice using immunohistochemistry. The results showed that, compared to the sh-NC group, the sh-SIRT1 group had significantly lower expressions of SIRT1, FOXO3, and LC3B proteins, and significantly higher expression of p62 protein. Conversely, the oe-SIRT1 group had significantly higher expressions of SIRT1, FOXO3, and LC3B proteins, and significantly lower expression of p62 protein compared to the oe-NC group (Fig. 9A–D). RT-qPCR and Western blot analyses revealed that, compared to the sh-NC group, the sh-SIRT1 group had significantly lower expressions of BNIP3, PINK1, and PRKN and their corresponding proteins in the tumor tissues. Conversely, the oe-SIRT1 group exhibited significantly higher expressions of these mRNAs and proteins compared to the oe-NC group (Fig. 9E, F).

Additionally, immunofluorescence staining was used to detect the localization of PINK1 and Parkin proteins in different treatment groups' xenograft tumors. The results confirmed that silencing SIRT1 inhibited the expression and co-localization of PINK1 and Parkin proteins, whereas overexpressing SIRT1 promoted their expression and co-localization (Fig. 9G).

These findings are consistent with the results of in vitro experiments and further confirm that the overexpression of SIRT1 can promote EC growth in vivo through the BNIP3/PINK1/Parkin signaling pathway and induce hormone resistance by upregulating mitophagy.

### Combination of MA and CQ enhances hormone resistance via mitophagy

**Fig. 10:**
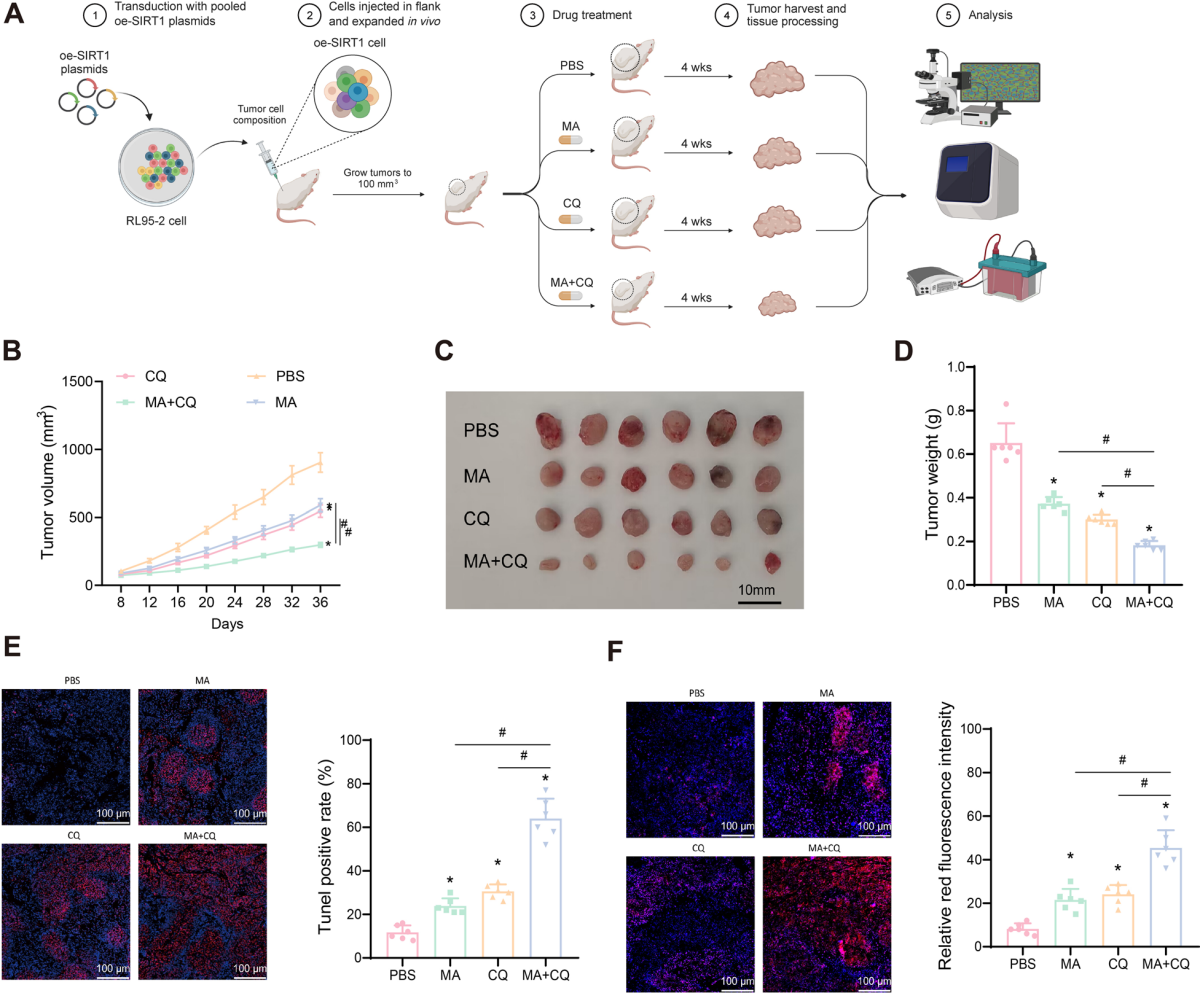
Overexpression of SIRT1 enhances tumor growth by inducing cell autophagy. **A** Schematic diagram of the animal experiment process (n = 6); **B** Line graph showing the growth of subcutaneously transplanted tumors in mice from day 8 to day 36 (n = 6); **C** Anatomical diagram of subcutaneously transplanted tumors in mice on day 36 (n = 6); **D** Statistics of tumor weight in mice with subcutaneously transplanted tumors on day 36 (n = 6); **E** TUNEL staining to detect apoptosis of tumor cells in mice in each group (n = 6); **F** MitoSOX immunofluorescence staining to detect the generation of ROS in tumor tissues of mice in each group (n = 6). Data are presented as mean ± SD, with 6 nude mice in each group. *p < 0.05 compared to the PBS group; ^#^*p* < 0.05 between the two groups

To further validate that SIRT1 overexpression induces mitophagy through FOXO3 deacetylation and enhances hormone resistance in EC cells, we constructed a SIRT1-overexpressing cell xenograft mouse model. When the average tumor volume reached 100 mm^3^, daily intraperitoneal drug administration began, with tumor volumes measured every 4 days (Fig. 10A). After 28 days of treatment, the average tumor volume in the MA group was (1283.33 ± 74.53) mm^3^, and in the CQ group it was (1166.67 ± 93.83) mm^3^, both significantly smaller than that of the PBS group (1833.33 ± 134.37) mm^3^. However, the tumor volume in the MA + CQ group was significantly reduced to (516.67 ± 38.05) mm^3^, compared to all other groups (Fig. 10B). After 28 days of continuous treatment, tumors were dissected, photographed, and weighed. The results showed that tumor volume and weight were significantly reduced in the MA and CQ groups compared to the PBS group, and further reduced in the MA + CQ group compared to the MA and CQ groups (Fig. 10C-D).

TUNEL assay results indicated that apoptosis in tumor cells increased in the MA and CQ groups compared to the PBS group, and further increased in the MA + CQ group compared to the MA and CQ groups (Fig. 10E). MitoSOX immunofluorescence staining showed a significant increase in ROS production in the tumors of the MA and CQ groups, with even higher ROS levels in the MA + CQ group (Fig. 10F).

**Fig. 11:**
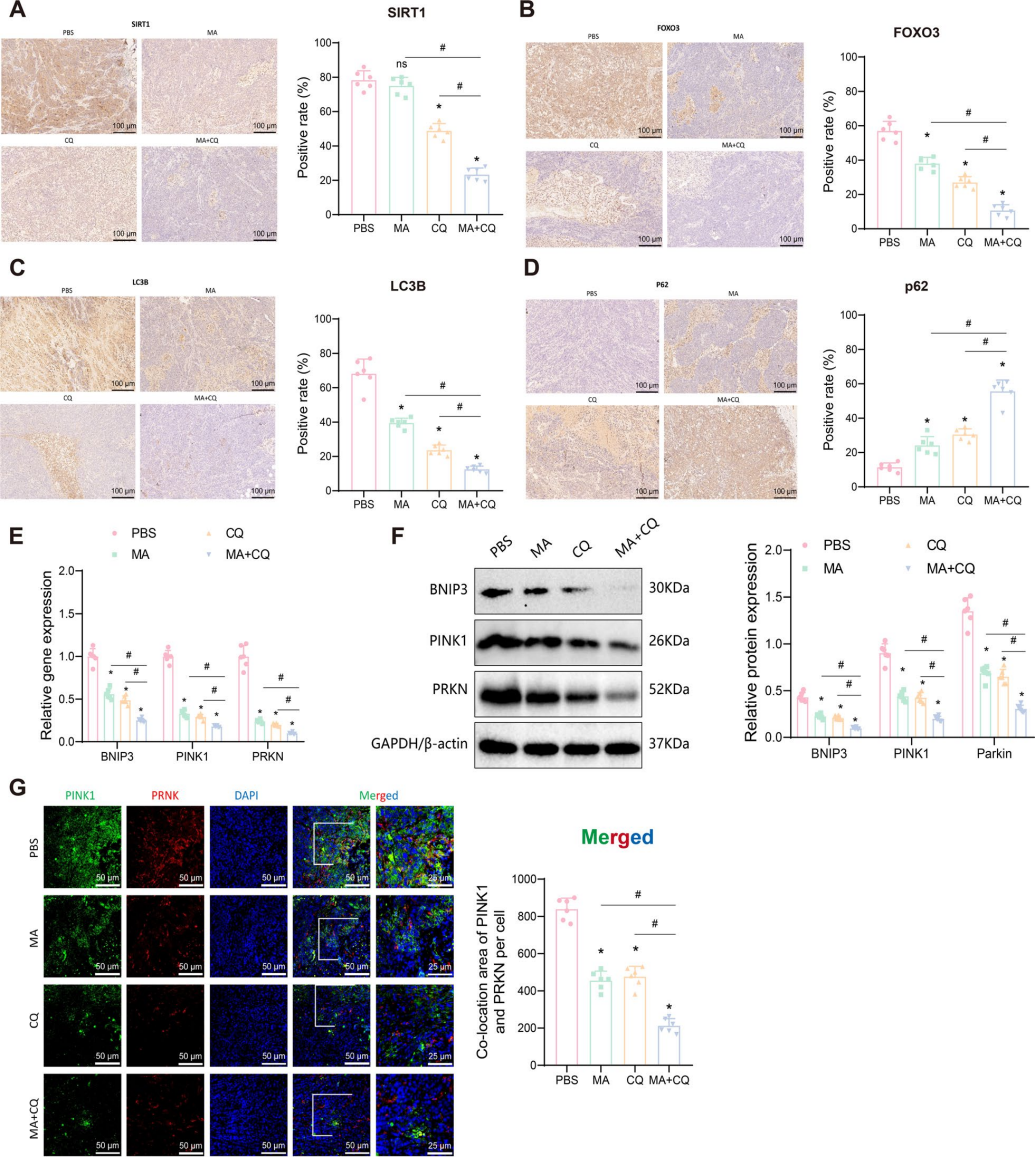
Overexpression of SIRT1 induces mitophagy and causes EC hormone Resistance. **A** Immunohistochemical staining to detect the expression changes of SIRT1 protein in tumor tissues of mice in each group (n = 6); **B** Immunohistochemical staining to detect the expression changes of FOXO3 protein in tumor tissues of mice in each group (n = 6); **C** Immunohistochemical staining to detect the expression changes of LC3B protein in tumor tissues of mice in each group (n = 6); **D** Immunohistochemical staining to detect the expression changes of p62 protein in tumor tissues of mice in each group (n = 6); **E** RT-qPCR to detect the expression of BNIP3, PINK1, and PRKN mRNA in tumor tissues of mice in each group; **F** Western blot to detect the expression of BNIP3, PINK1, and Parkin protein in tumor tissues of mice in each group; **G** Immunofluorescence staining to detect the expression and colocalization of PINK1 and Parkin proteins in tumor tissues of mice in each group. Data are presented as mean ± SD. *p < 0.05 compared to the PBS group; ^#^*p* < 0.05 between the two groups

Immunohistochemistry results revealed no significant change in SIRT1 protein expression in the MA group compared to the PBS group, while SIRT1 protein expression decreased in the CQ group. Both the MA and CQ groups exhibited reduced FOXO3 and LC3B protein expression and increased p62 protein expression. These changes were more pronounced in the MA + CQ group (Fig. 11A-D). RT-qPCR and Western blot analyses confirmed that BNIP3, PINK1, and PRKN mRNA and protein expression were significantly lower in the MA and CQ groups compared to the PBS group, and even further reduced in the MA + CQ group (Fig. 11E-F).

Additionally, immunofluorescence staining demonstrated that MA and CQ treatments inhibited the expression and co-localization of PINK1 and Parkin proteins, with an even greater inhibition observed in the MA + CQ treatment group (Fig. 11G).

These results further confirm that overexpression of SIRT1 in EC cells promotes tumor growth and hormone resistance through the BNIP3/PINK1/Parkin signaling pathway by upregulating mitophagy. The combination of MA and CQ effectively inhibits these processes, highlighting the potential therapeutic strategy for overcoming hormone resistance in EC.

## Discussion

EC is a highly prevalent gynecological malignancy. Current treatment options for EC include surgery, radiotherapy, chemotherapy, and hormone therapy (Rütten et al. 2021). However, the effectiveness of these treatments is often limited by the problem of hormone resistance, which significantly hampers treatment efficacy (Gordhandas et al. 2023). Although hormone resistance may not directly impact the mechanisms of radiotherapy or chemotherapy, it can indirectly affect these treatments through multidrug resistance (MDR) (Duan et al. 2023). Therefore, understanding the pathogenesis of EC, particularly the molecular mechanisms related to hormone resistance, is crucial for improving treatment strategies for EC.

Interestingly, SIRT1 and FOXO3 have been shown to play critical roles in various types of cancer, typically influencing tumor progression by regulating cell growth, apoptosis, and autophagy (Patra et al. 2023; Orea-Soufi et al. 2022). In our study on EC cells, we observed significant alterations in the expression levels of SIRT1 and FOXO3, consistent with reports in other cancer types. Furthermore, our research suggests that SIRT1 and FOXO3 in EC can function through the common regulation of mitophagy, a mechanism that has been rarely reported before. Previous studies have suggested that SIRT1 can deacetylate various target proteins, including FOXO3 (Yao et al. 2022). In our study, a series of experiments, including Co-IP, provided clear evidence of a direct PPI between SIRT1 and FOXO3. This interaction appears to play a crucial role in autophagy and cell survival in EC cells, further emphasizing the roles of both proteins in this type of cancer.

Mitophagy, the process of eliminating damaged mitochondria within cells, has been recognized as a crucial process, and previous studies have shown that abnormalities in mitophagy can lead to various diseases, including cancer (Springer and Macleod 2016). Our research results demonstrate that SIRT1 and FOXO3 can regulate mitophagy in EC cells, which differs slightly from reports in other cancers. We found that mitophagy promotes cell growth, proliferation, migration, and invasion while inhibiting apoptosis in EC. Due to the significant roles of SIRT1 and FOXO3 in EC, they have emerged as potential therapeutic targets. Our research further emphasizes the role of FOXO3 as another important target. Future strategies may involve combination therapies targeting both SIRT1 and FOXO3, offering new hope for EC patients. The techniques we employed, such as high-throughput sequencing and bioinformatics analysis, greatly assisted in investigating the roles of SIRT1 and FOXO3 in EC. However, we must acknowledge the limitations of these techniques. For example, while high-throughput sequencing provides abundant data, it requires more intricate analysis tools. Although our results align with previous research, further validation experiments are still necessary.

Our study has opened up novel avenues for exploring the role of SIRT1 and FOXO3 in EC. Future research can delve into the mechanisms underlying their interactions and how they regulate other cancer-related signaling pathways. Additionally, we hope to develop more specific and efficient drugs that target SIRT1 and FOXO3 for therapeutic purposes. In summary, our study reveals the potential importance of SIRT1 and FOXO3 in EC through their regulation of mitophagy. Overexpression of SIRT1 in EC cells promotes FOXO3 protein deacetylation, enhances FOXO3 expression, stimulates BNIP3 transcription, and triggers cell mitophagy via the PINK1/Parkin pathway, resulting in increased cell growth, proliferation, migration, and invasion while suppressing apoptosis. This finding lays the groundwork for further exploration of both proteins as potential therapeutic targets. We anticipate that future research may provide more effective treatment approaches for EC patients.

In recent years, there has been growing interest in understanding the regulatory role of SIRT1 in various cancers, including EC (Chen et al. 2021; Alves-Fernandes and Jasiulionis 2019). In this study, we found that SIRT1 plays a critical role in the growth of EC, which is consistent with previous reports on SIRT1 in other cancers. Early studies primarily focused on the relationship between SIRT1 and cellular aging, survival, and metabolism regulation, while its specific function in certain cancers remains controversial (Carafa et al. 2019; Deng 2009). Our findings demonstrate a significant difference in the tumor growth rate of EC when the activity of SIRT1 is modulated, thus emphasizing its key role in cancer progression.

A notable aspect of this study was the investigation of the interaction between SIRT1 and hormone response. While reports on the association between SIRT1 and hormone treatment response in other cancers exist (Wang et al. 2018), we have made the novel discovery that changes in SIRT1 activity significantly affect the tumor's response to hormones in EC. This finding aligns with earlier suggestions that SIRT1 might regulate the hormone response (Wang et al. 2018). Importantly, we provide direct in vivo evidence for this viewpoint. In our investigation of the expression of FOXO3, LC3B, and other related proteins under SIRT1 regulation, we observed significant alterations in cellular localization and expression of these proteins upon SIRT1 silencing or overexpression. These findings further support the crucial role of SIRT1 in EC development.

Extensive literature has reported the influence of SIRT1 on mitochondrial function (Tang 2016). In our study, we compared the effects of SIRT1 activation and inhibition on MMP and ROS production, providing additional evidence for the central role of SIRT1 in maintaining mitochondrial health. Importantly, these findings are not only consistent with in vitro cell experiments but have also been validated in an in vivo EC model. Autophagy and cell apoptosis are two critical determinants of cell fate (Das et al. 2021). In this study, we observed significant effects of SIRT1 activity modulation on these two processes. Consistent with most of the literature, our results demonstrate that SIRT1 activation enhances autophagy while reducing cell apoptosis. These findings provide compelling in vivo evidence for the potential application of SIRT1 in cancer treatment.

In our study, we have revealed a crucial regulatory role of the PPI between SIRT1 and FOXO3 in the development of EC. This interaction may involve the deacetylation modification of FOXO3 by SIRT1, which in turn affects its transcriptional activity and cellular localization. It is noteworthy that the FOXO3-associated signaling pathways have been recognized as therapeutic targets for various cancers (Orea-Soufi et al. 2022), and our research findings provide a novel mechanistic understanding of SIRT1 as an upstream regulatory factor. Furthermore, this PPI offers valuable clues for designing novel therapeutic strategies targeting EC, such as intervening with the interaction between SIRT1 and FOXO3 using drug innovations.

## Conclusion

Overall, our study provides a series of novel insights into the role of SIRT1 in EC, indicating its central regulatory role in the development of this cancer. Overexpression of SIRT1 in EC cells promotes FOXO3 protein deacetylation, resulting in increased FOXO3 expression and BNIP3 protein transcription. Additionally, it stimulates cell mitophagy through the PINK1/Parkin pathway, thereby promoting in vitro growth, migration, and invasion of EC cells, inhibiting cell apoptosis, and facilitating the growth of EC cell xenografts in vivo. Moreover, it promotes hormone resistance. Meanwhile, our research also provides important in vivo evidence and potential drug targets for novel therapeutic strategies for EC. Future studies need to further explore the exact mechanisms of SIRT1 and FOXO3 in EC, as well as how to effectively utilize these findings for clinical treatment.

## Supplementary Information

Additional file 1: Figure S1. Schematic representation of the potential molecular mechanisms. SIRT1 promotes the deacetylation of FOXO3 protein in EC cells, enhancing FOXO3 expression, which in turn promotes the transcription of BNIP3 protein. BNIP3, through the PINK1/Parkin pathway, facilitates mitophagy, leading to increased cell growth, proliferation, migration, and invasion of EC cells in vitro. It also inhibits apoptosis and enhances tumor growth and hormone resistance in vivo

Additional file 2: Figure S2. Key pathways revealing the relationship between EC and mitophagy based on bioinformatics analysis. **A** Workflow of high-throughput transcriptional sequencing analysis; **B** Kaplan-Meier survival curve analysis of the TCGA database for the relationship between the expression of SIRT1, FOXO3, and PINK1 genes and overall survival in EC; **C** Bubble chart of the GO functional enrichment analysis results for 76 EARGs; **D** Circle chart of the GO functional enrichment analysis results for 76 EARGs; **E** Illustration of the FoXO signaling pathway in the KEGG pathway enrichment analysis results.

Additional file 3: Figure S3. Validation of silencing efficiency of SIRT1 and FOXO3 mRNA. **A** Changes in the expression of SIRT1 mRNA after silencing SIRT1 with different sequences as detected by RT-qPCR in RL95-2 cells; **B** Changes in the expression of SIRT1 protein after silencing SIRT1 mRNA with different sequences as detected by Western blot in RL95-2 cells; **C** Changes in the expression of FOXO3 mRNA after silencing FOXO3 mRNA with different sequences as detected by RT-qPCR in RL95-2 cells; **D** Changes in the expression of FOXO3 protein after silencing FOXO3 mRNA with different sequences as detected by Western blot in RL95-2 cells. Data are presented as mean ± SD, with each cellular experiment repeated three times. *p < 0.05 compared to the sh-NC group.

Additional file 4: Figure S4. Further validation of the regulation of mitophagy in EC cells by overexpression of SIRT1. **A** CCK-8 assay assessing the effect of autophagy inhibitor CQ on the viability of oe-SIRT1 cells; **B** EdU experiment evaluating the effect of CQ on the proliferation capability of oe-SIRT1 cells, with EdU-positive cells shown in pink color and EdU-negative cells shown in blue color; **C** Colony formation assay examining the impact of CQ on the colony formation ability of oe-SIRT1 cells; **D** Transwell invasion experiment determining the influence of CQ on the migration and invasion ability of oe-SIRT1 cells; **E** Flow cytometry analysis investigating the apoptosis status of oe-SIRT1 cells after treatment with CQ; **F** JC-1 staining experiment to measure the changes in MMP of oe-SIRT1 cells after treatment with CQ; **G** TEM to assess the mitochondrial ultrastructure; **H** MitoSOX immunofluorescence staining investigating the alteration in ROS production in oe-SIRT1 cells after treatment with CQ; **I** Immunofluorescence staining examining the transfection of GFP-LC3B plasmid in oe-SIRT1 cells after treatment with CQ; **J** RT-qPCR analysis to measure the expression changes of MAP1LC3A and SQSTM1 mRNA in oe-SIRT1 cells after treatment with CQ; **K** Western blot analysis to measure the expression changes of LC3AI, LC3AII, and p62 proteins in oe-SIRT1 cells after treatment with CQ (Note: LC3AI and LC3AII bands are presented on a single gel image). Data are presented as mean ± SD, with each cellular experiment repeated three times. *p < 0.05 compared to the oe-SIRT1 + DMSO group.

Additional file 5.

## Data Availability

All data can be provided as needed.
