# Supplementary figures and images for: SIRT1-mediated deacetylation of FOXO3 enhances mitophagy and drives hormone resistance in endometrial cancer
Source: Mol Med. 2024 Sep 12;30:147. doi: 10.1186/s10020-024-00915-7 (PMC11391609; doi:10.1186/s10020-024-00915-7)

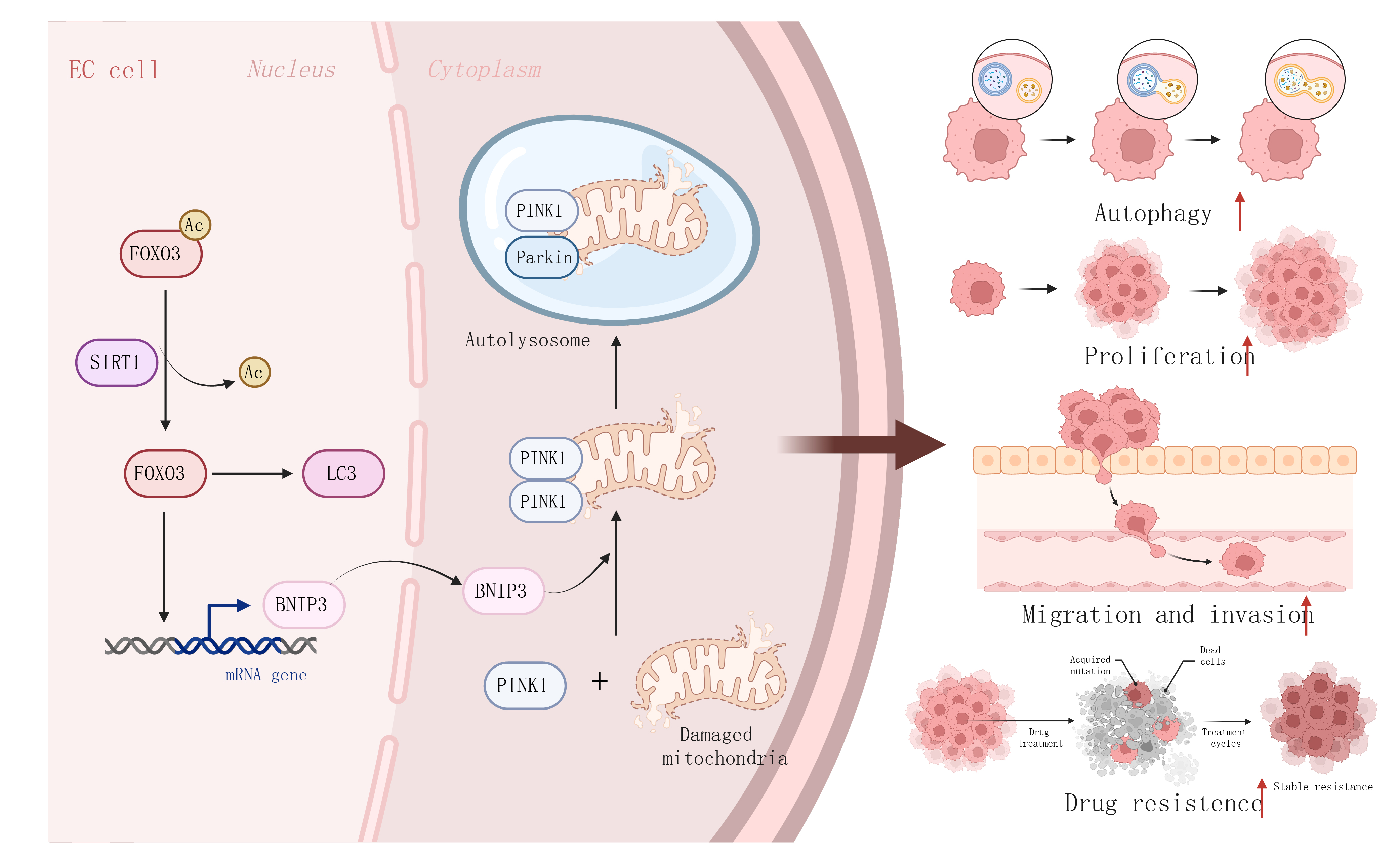

Supplement: Supplementary file 1 — Additional file 1: Figure S1. Schematic representation of the potential molecular mechanisms. SIRT1 promotes the deacetylation of FOXO3 protein in EC cells, enhancing FOXO3 expression, which in turn promotes the transcription of BNIP3 protein. BNIP3, through the PINK1/Parkin pathway, facilitates mitophagy, leading to increased cell growth, proliferation, migration, and invasion of EC cells in vitro. It also inhibits apoptosis and enhances tumor growth and hormone resistance in vivo [file 10020_2024_915_MOESM1_ESM.jpg]

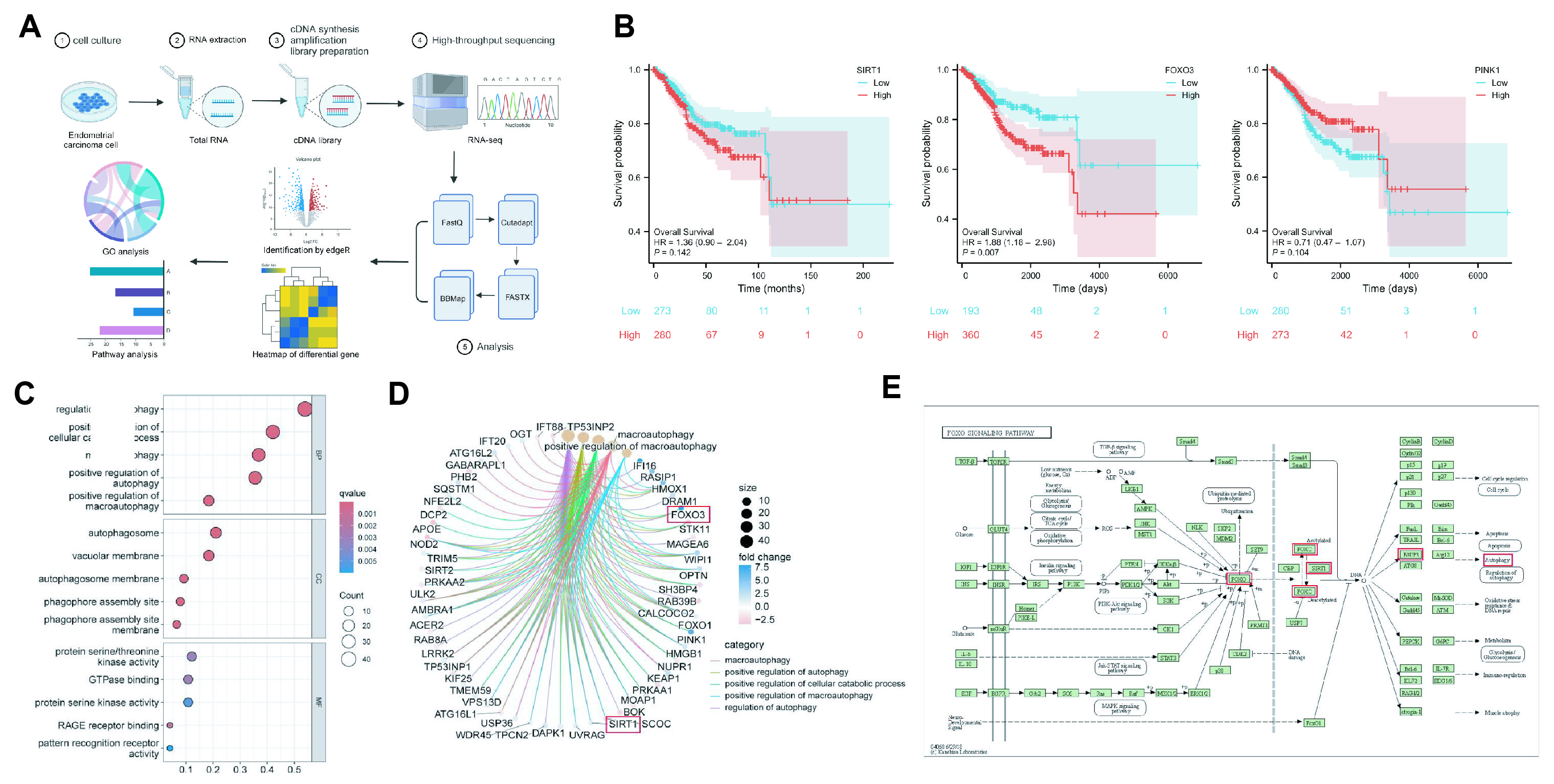

Supplement: Supplementary file 2 — Additional file 2: Figure S2. Key pathways revealing the relationship between EC and mitophagy based on bioinformatics analysis. A Workflow of high-throughput transcriptional sequencing analysis; B Kaplan-Meier survival curve analysis of the TCGA database for the relationship between the expression of SIRT1, FOXO3, and PINK1 genes and overall survival in EC; C Bubble chart of the GO functional enrichment analysis results for 76 EARGs; D Circle chart of the GO functional enrichment analysis results for 76 EARGs; E Illustration of the FoXO signaling pathway in the KEGG pathway enrichment analysis results. [file 10020_2024_915_MOESM2_ESM.jpg]

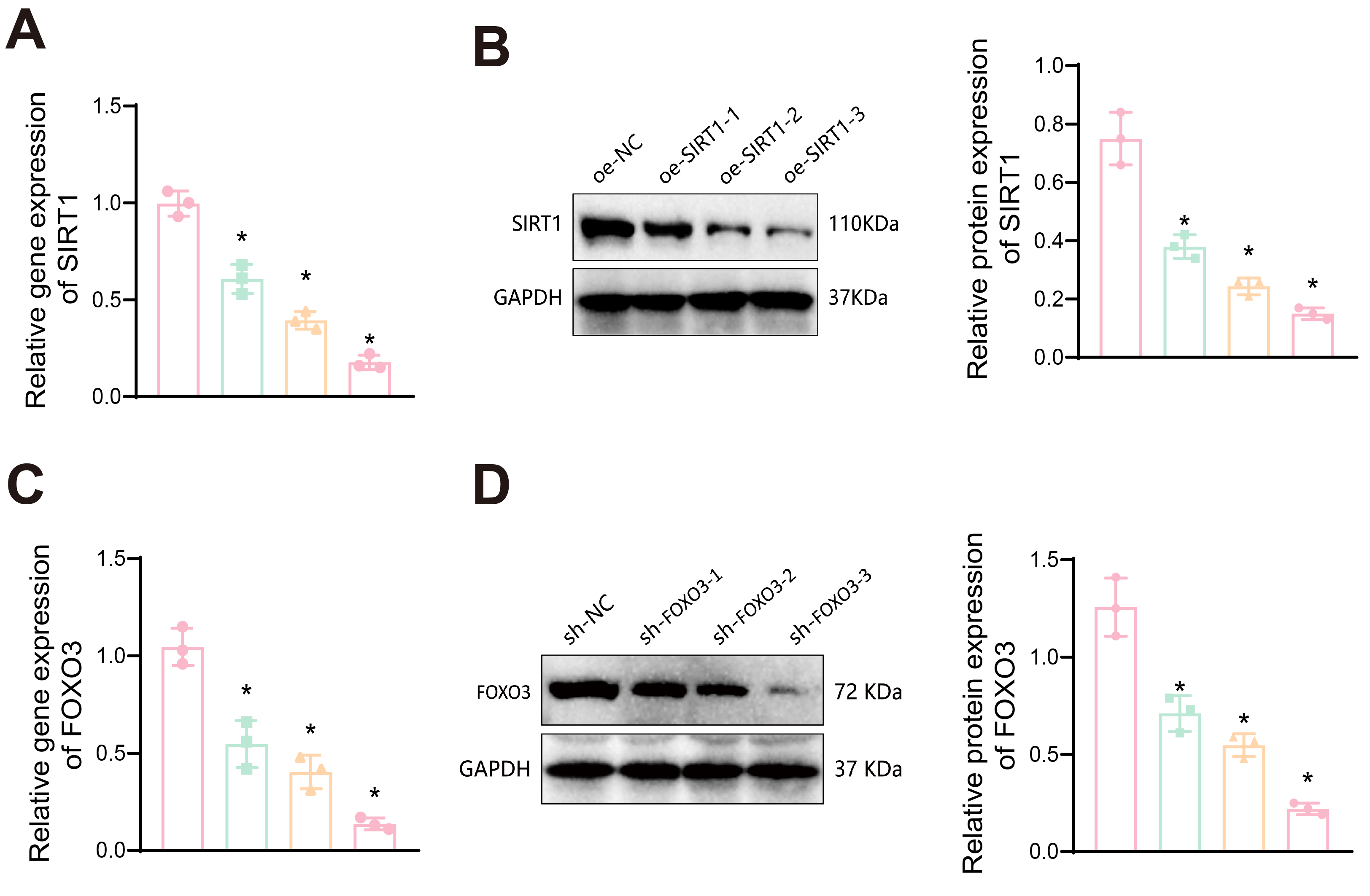

Supplement: Supplementary file 3 — Additional file 3: Figure S3. Validation of silencing efficiency of SIRT1 and FOXO3 mRNA. A Changes in the expression of SIRT1 mRNA after silencing SIRT1 with different sequences as detected by RT-qPCR in RL95-2 cells; B Changes in the expression of SIRT1 protein after silencing SIRT1 mRNA with different sequences as detected by Western blot in RL95-2 cells; C Changes in the expression of FOXO3 mRNA after silencing FOXO3 mRNA with different sequences as detected by RT-qPCR in RL95-2 cells; D Changes in the expression of FOXO3 protein after silencing FOXO3 mRNA with different sequences as detected by Western blot in RL95-2 cells. Data are presented as mean ± SD, with each cellular experiment repeated three times. *p < 0.05 compared to the sh-NC group. [file 10020_2024_915_MOESM3_ESM.jpg]

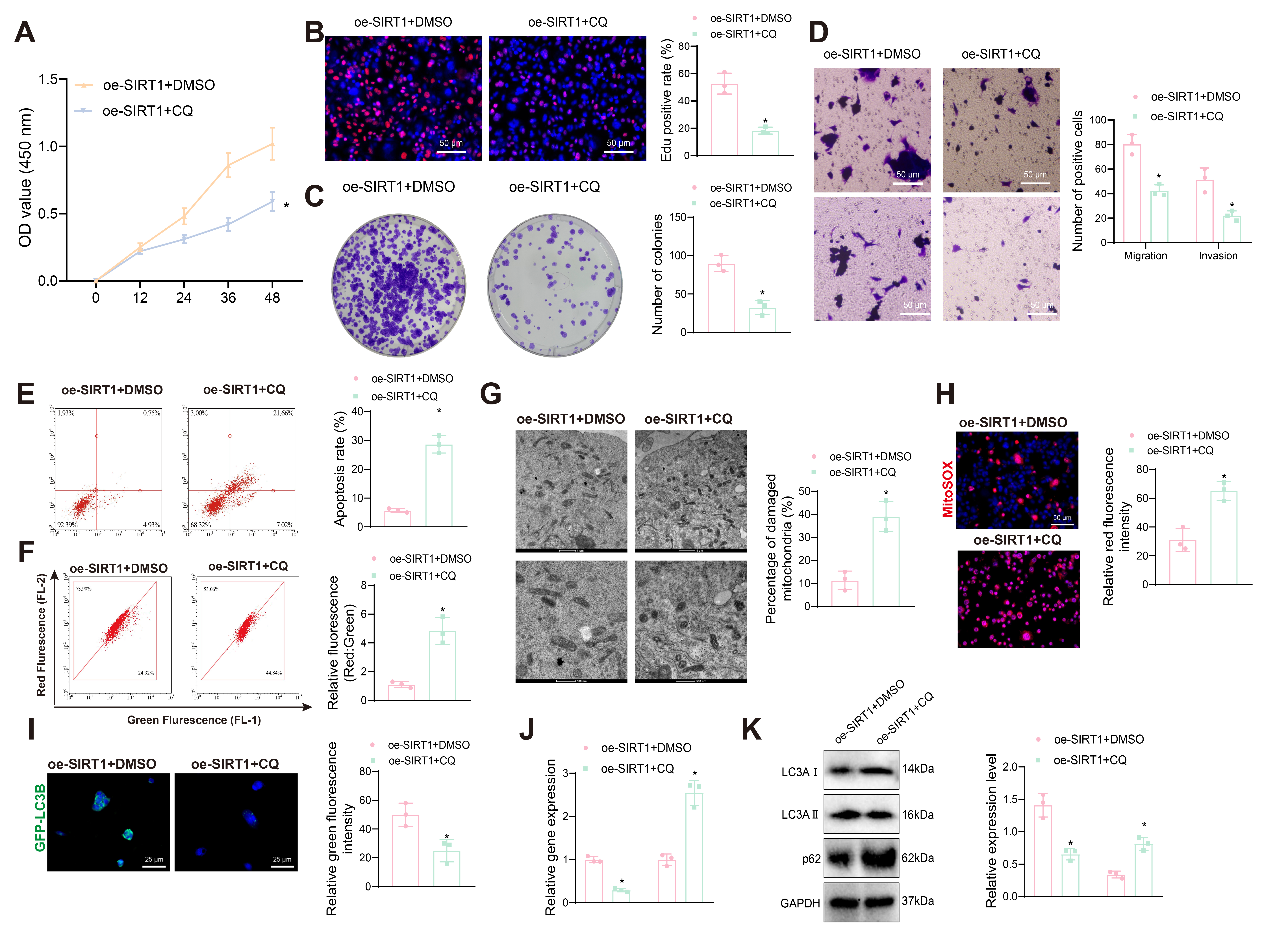

Supplement: Supplementary file 4 — Additional file 4: Figure S4. Further validation of the regulation of mitophagy in EC cells by overexpression of SIRT1. A CCK-8 assay assessing the effect of autophagy inhibitor CQ on the viability of oe-SIRT1 cells; B EdU experiment evaluating the effect of CQ on the proliferation capability of oe-SIRT1 cells, with EdU-positive cells shown in pink color and EdU-negative cells shown in blue color; C Colony formation assay examining the impact of CQ on the colony formation ability of oe-SIRT1 cells; D Transwell invasion experiment determining the influence of CQ on the migration and invasion ability of oe-SIRT1 cells; E Flow cytometry analysis investigating the apoptosis status of oe-SIRT1 cells after treatment with CQ; F JC-1 staining experiment to measure the changes in MMP of oe-SIRT1 cells after treatment with CQ; G TEM to assess the mitochondrial ultrastructure; H MitoSOX immunofluorescence staining investigating the alteration in ROS production in oe-SIRT1 cells after treatment with CQ; I Immunofluorescence staining examining the transfection of GFP-LC3B plasmid in oe-SIRT1 cells after treatment with CQ; J RT-qPCR analysis to measure the expression changes of MAP1LC3A and SQSTM1 mRNA in oe-SIRT1 cells after treatment with CQ; K Western blot analysis to measure the expression changes of LC3AI, LC3AII, and p62 proteins in oe-SIRT1 cells after treatment with CQ (Note: LC3AI and LC3AII bands are presented on a single gel image). Data are presented as mean ± SD, with each cellular experiment repeated three times. *p < 0.05 compared to the oe-SIRT1 + DMSO group. [file 10020_2024_915_MOESM4_ESM.jpg]
