# Supplementary material for: SIRT1-mediated deacetylation of FOXO3 enhances mitophagy and drives hormone resistance in endometrial cancer
Source: Mol Med. 2024 Sep 12;30:147. doi: 10.1186/s10020-024-00915-7 (PMC11391609; doi:10.1186/s10020-024-00915-7)
Supplement: Supplementary file 5 — Additional file 5. [file 10020_2024_915_MOESM5_ESM.docx]

**Table S1.** **Lentiviral transfection gene silencing sequences**

| **Name** | **shRNA Sequences (5'- 3')** |
| --- | --- |
| sh-NC | 5'- CCTAAGGTTAAGTCGCCCTCG -3' |
| sh-SIRT1-1 | 5'- GCACAGATCCTCGAACAATTC -3' |
| sh-SIRT1-2 | 5'- GCAACTATACCCAGAACATAG -3' |
| sh-SIRT1-3 | 5'- GCGGGAATCCAAAGGATAATT -3' |
| sh-FOXO3-1 | 5'- CCGGCACCATGAATCTGAATG -3' |
| sh-FOXO3-2 | 5'- GGAACTTCACTGGTGCTAAGC -3' |
| sh-FOXO3-3 | 5'- ATGGACAATAGCAACAAGTATAC -3' |

**Table S2. RT-qPCR** **primer sequence**

| **Gene Name** | **Primer Sequence** |
| --- | --- |
| SIRT1 (Human) | Forward: 5'- CTACTGGCCTGAGGTTGAGG -3' |
|  | Reverse: 5'- GGACGGAGGAAAAGAGCGAA -3' |
| FOXO3 (Human) | Forward: 5'- ATCCTGTACGTGGCCCCT -3' |
|  | Reverse: 5'- CTCCTCGGGGATCATGGAGT -3' |
| MAP1LC3A (Human) | Forward: 5'- CATCTGCCCCTCACCCAC -3' |
|  | Reverse: 5'- ATACACCACGTGGAGACCGA -3' |
| SQSTM1 (Human) | Forward: 5'- CATTGCGGAGCCTCATCTCCT -3' |
|  | Reverse: 5'- AAGTCCCCGTCCTCATCCTTT -3' |
| PINK1 (Human) | Forward: 5'- CCATCTGGTTCAACAGGGCA -3' |
|  | Reverse: 5'- AAATCTGCGATCACCAGCCA -3' |
| PRKN (Human) | Forward: 5'- GACAGCAGGAAGGACTCACC -3' |
|  | Reverse: 5'- GCTGCACTGTACCCTGAGTT -3' |
| BNIP3 (Human) | Forward: 5'- CAACCTCCACCAGCACCTTT -3' |
|  | Reverse: 5'- GGCCACCCCAGGATCTAACA -3' |
| GAPDH (Human) | Forward: 5'- TGCAACCGGGAAGGAAATGA -3' |
|  | Reverse: 5'- GCATCACCCGGAGGAGAAAT -3' |

**Table S3. Western blot antibody information**

| **Target Name** | **Manufacturer** | **Item Number** | **Dilution Ratio** |
| --- | --- | --- | --- |
| SIRT1 (Human) | Abcam | ab76039 | 1:1000 |
| FOXO3 (Human) | Abcam | ab314007 | 1:1000 |
| LC3B (Human) | Abcam | ab192890 | 1:2000 |
| MAP1LC3A (Human) | Abclonal | A12319 | 1:1000 |
| p62 (Human) | Abcam | ab207305 | 1:1000 |
| PINK1 (Human) | Abcam | ab216144 | 1:1000 |
| Parkin (Human) | Abclonal | A0968 | 1:20000 |
| BNIP3 (Human) | Abcam | ab109362 | 1:2000 |
| GAPDH (Human) | Abcam | ab9485 | 1:1000 |

Note: Antibodies purchased from Abcam, UK, or Abclonal, Wuhan China.

**Table S4. CytoHubba gene network topology properties**

| **Gene Name** | **Degree** | **EPC** | **MCC** | **MNC** | **DMNC** |
| --- | --- | --- | --- | --- | --- |
| SIRT1 | 30 | 25.016 | 63 | 14 | 0.47297 |
| FOXO3 | 24 | 24.539 | 83 | 9 | 0.85919 |
| PINK1 | 22 | 24.729 | 74 | 9 | 0.85919 |
| FOXO1 | 20 | 24.661 | 42 | 10 | 0.55867 |
| SQSTM1 | 18 | 23.816 | 24 | 9 | 0.52506 |
| AMBRA1 | 16 | 22.27 | 39 | 7 | 0.80493 |
| ULK2 | 14 | 23.834 | 34 | 7 | 0.73175 |
| GABARAPL1 | 14 | 23.755 | 60 | 7 | 0.95128 |
| UVRAG | 14 | 23.874 | 66 | 7 | 1.02445 |
| CALCOCO2 | 14 | 23.695 | 38 | 7 | 0.80493 |
| WIPI1 | 12 | 11.657 | 6 | 2 | 0.61557 |
| ATG16L1 | 12 | 22.963 | 28 | 6 | 0.76079 |
| STK11 | 12 | 21.376 | 31 | 5 | 1.03722 |
| OPTN | 12 | 21.784 | 36 | 6 | 0.95098 |
| PRKAA1 | 10 | 22.586 | 48 | 5 | 1.16687 |
| PRKAA2 | 10 | 18.797 | 25 | 4 | 1.13679 |
| NFE2L2 | 10 | 19.531 | 6 | 3 | 0.61795 |
| RAB8A | 8 | 18.863 | 24 | 4 | 1.13679 |
| ATG16L2 | 8 | 17.819 | 5 | 3 | 0.61795 |
| TLR4 | 8 | 19.465 | 5 | 3 | 0.61795 |
| HMOX1 | 8 | 19.565 | 8 | 4 | 0.75786 |
| KEAP1 | 6 | 18.423 | 6 | 3 | 0.92693 |
| TP53INP1 | 6 | 15.324 | 3 | 1 | 0 |
| TRIM5 | 4 | 15.712 | 2 | 2 | 0.61557 |
| IFT20 | 4 | 12.332 | 2 | 1 | 0 |
| IFT88 | 4 | 14.889 | 2 | 2 | 0.61557 |
| SIRT2 | 4 | 15.384 | 2 | 2 | 0.61557 |
| LRRK2 | 4 | 10.791 | 2 | 1 | 0 |
| HMGB1 | 4 | 6.311 | 2 | 2 | 0.61557 |
| S100A9 | 4 | 6.652 | 2 | 2 | 0.61557 |
| MYO6 | 4 | 8.606 | 2 | 1 | 0 |
| OFD1 | 4 | 9.45 | 2 | 1 | 0 |
| RAB39B | 4 | 15.679 | 2 | 2 | 0.61557 |
| NOD2 | 4 | 16.007 | 2 | 2 | 0.61557 |
| PHB2 | 4 | 14.177 | 2 | 2 | 0.61557 |
| APOE | 2 | 5.312 | 1 | 1 | 0 |
| TMEM59 | 2 | 9.705 | 1 | 1 | 0 |
| FNIP1 | 2 | 7.822 | 1 | 1 | 0 |
| OGT | 2 | 9.876 | 1 | 1 | 0 |
| TP53INP2 | 2 | 9.844 | 1 | 1 | 0 |
| GBP1 | 2 | 9.817 | 1 | 1 | 0 |
| LGALS3 | 2 | 4.966 | 1 | 1 | 0 |
| NUPR1 | 2 | 4.87 | 1 | 1 | 0 |

Note: EPC: Edge penetration component; MNC: Maximum neighborhood component; DMNC: Density of maximum neighborhood components; MCC: Maximum group centrality
